# Bicontinuous Interconnected Porous Biomaterials for Tissue Engineering and Regeneration

**DOI:** 10.1002/smsc.202500207

**Published:** 2025-08-28

**Authors:** Aihik Banerjee, Anjana Khanal, Prince D. Okoro, Shankar P. Kharal, Kevin Dalsania, Baishali Kanjilal, Shiril B. Iragavarapu, Yiqing Chen, Janitha M. Unagolla, Huinan H. Liu, Joshua T. Morgan, Robert P. Hesketh, Arash Pezhouman, Reza Ardehali, Bahman Anvari, Martin F. Haase, Iman Noshadi

**Affiliations:** ^1^ Department of Bioengineering University of California, Riverside Riverside CA 92521 United States; ^2^ Department of Chemical Engineering Rowan University Glassboro NJ 08028 United States; ^3^ Materials Science and Engineering Program University of California, Riverside Riverside CA 92521 United States; ^4^ Department of Medicine Division of Cardiology Baylor College of Medicine Houston TX 77030 United States; ^5^ Van't Hoff Laboratory of Physical and Colloid Chemistry Department of Chemistry Debye Institute for Nanomaterials Science Utrecht University 3584 Utrecht CS The Netherlands

**Keywords:** bicontinuous materials, human induced pluripotent stem cells, interconnected porosity, polyethylene glycol diacrylate, solvent transfer‐induced phase separation

## Abstract

Biomaterials mimicking natural extracellular matrix are necessary to create an optimal microenvironment for cell adhesion, migration, proliferation, and differentiation. These scaffolds must possess bicontinuous interconnected porosity to ensure the effective exchange of oxygen, nutrients, and metabolic waste, which are crucial for developing functional tissues. Here, a novel bicontinuous interfacially jammed emulsion (BIJEL)‐Integrated PORous Engineered System (BIPORES) is developed to confer bioinert synthetic polyethylene glycol diacrylate (PEGDA) with unique bicontinuous interconnected porosity and surface topography. This platform is fabricated through controlled phase separation and interfacial stabilization of two continuous phases by nanoparticles. Functional validation using human mesenchymal stem cells, and human induced pluripotent stem cells‐derived cardiomyocytes and cardiac fibroblasts, reveals outstanding cell attachment, growth, proliferation, and/or differentiation within tissue‐scale BIPORES scaffolds. These findings indicate that bicontinuous interconnected porosity with negative Gaussian curvature in the BIPORES scaffolds plays a key role in organ‐scale tissue engineering and regeneration.

## Graphical abstract

Solvent transfer‐induced phase separation transforms bioinert polyethylene glycol diacrylate into bioactive scaffolds featuring bicontinuous interconnected porosity, with negative Gaussian curvature, and unique topographical patterning. These attributes enable stem cell attachment, proliferation, and differentiation without basement membrane matrix proteins in vitro and promote tissue integration without immune rejection in vivo. This platform represents a promising strategy for organ‐scale tissue engineering and regeneration.

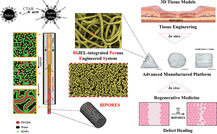

## 1 Introduction

Engineered tissues can function as models for testing new drugs or studying diseases. However, translating tissue engineering into clinically viable solutions faces challenges, particularly in creating functional engineered tissues of appropriate size. Critical obstacles are the limited diffusion of oxygen and nutrients and inadequate vascular integration within dense tissue constructs.^[^ ^1^, ^2^, ^3^ ^]^ A key strategy involves developing scaffolding materials with optimal pore characteristics to enhance oxygen and nutrient flow within engineered tissues and organ constructs. Hierarchical porous structures are essential, providing suitable surface characteristics for cell attachment, interconnected channels for cell migration, and optimal transport phenomena for oxygen and nutrition to promote vascularization.^[^ ^4^, ^5^, ^6^, ^7^ ^]^ Surface pores facilitate the delivery of biological cargo like proteins, genes, or cells.^[^ ^8^, ^9^ ^]^ Porous biomaterials also influence host immune responses and tissue integration. Patterned surfaces regulate macrophage activity and inflammation, while the presence of pores promotes vascularization and tissue ingrowth.^[^ ^10^, ^11^, ^12^, ^13^ ^]^

Several methods have been employed to make porous polymeric biomaterials, including porogen leaching/templating,^[^ ^14^, ^15^ ^]^ supercritical gas foaming,^[^ ^16^ ^]^ electrospinning,^[^ ^17^ ^]^ bioprinting,^[^ ^18^ ^]^ and emulsion polymerization.^[^ ^19^ ^]^ However, these techniques not only exhibit limitations in producing porous networks with desired interconnectivity, but they may also give rise to constricted pathways between void pockets that hinder the migration of cells, the diffusion of oxygen and nutrition, and the complete utilization of space. With respect to polymer selection, natural biopolymers, such as cellulose or collagen, offer high bioactivity, but are plagued by several limitations such as poor processability, batch‐to‐batch variability, limited tunability of mechanical, physical, or chemical properties, immunogenicity, and high production cost.^[^ ^20^, ^21^, ^22^ ^]^ Synthetic polymers such as polyethylene glycol diacrylate (PEGDA) can circumvent many of the limitations of natural polymers but lack cell adhesion sites. As such, a new class of materials comprising continuous interwoven fluid channels, termed bicontinuous materials, has gained popularity as a scaffold design choice due to their unique extracellular matrix (ECM)‐mimicking features.

Recent advancements in the field have introduced novel approaches aimed at simplifying the manufacturing process of bicontinuous interconnected porous materials. As an advanced manufacturing technique, Solvent Transfer‐Induced Phase Separation (STrIPS) allows for the generation of porous bicontinuous interfacially jammed emulsion (BIJEL)‐based materials from a wide range of immiscible liquid pairs, where a solvent facilitates temporary homogeneous mixing of the immiscible liquids meant to undergo phase separation.^[^ ^23^, ^24^, ^25^, ^26^, ^27^ ^]^ These ternary mixtures lead to a consistent sponge‐like surface architecture on a hierarchically structured scaffold with a porous substructure. Although STrIPS‐based materials have been used extensively in catalysis and separation processes,^[^ ^28^, ^29^, ^30^ ^]^ these materials are yet to be realized as optimal biomaterials for biomedical applications such as bioactive scaffolds and implantable materials. In this regard, STrIPS‐processed BIJEL‐Integrated PORous Engineered System (BIPORES) represents an innovative framework for creating biomaterials that possess an interconnected pore structure without any constriction, resulting in a uniformly negative Gaussian curvature along their inner surfaces.

Structurally, the BIPORES biomaterial is composed of interconnected fluid domains, where the fluids fully permeate, and the interfaces between them are occupied by a monolayer of nanoparticles. These interfaces have a distinctive hyperbolic curvature with a saddle‐like shape, where the curvature is negatively curved, and the mean curvature is balanced at zero.^[^ ^23^, ^24^ ^]^ By introducing nanoparticles into bicontinuous materials, it becomes possible not only to enhance their stability and functional characteristics but also to exert influence over the morphology, which can be tailored based on the specific type, geometry, and surface features of the nanoparticles.^[^ ^25^ ^]^ The hierarchical and asymmetric architecture of STrIPS‐BIPORES materials have many potential benefits for cell culture applications, such as the tunable porous surface layer serving as a substrate for cell attachment, an interpenetrating porous fiber center fulfilling the requirement of channels for nutritional exchange and metabolic waste drainage, the large surface area of the microporous walls can enhance cell attachment, growth, and proliferation, and the jammed nanoparticles may act as promoters of cellular functionalities such as differentiation of stem cells into specific lineages.^[^ ^31^, ^32^, ^33^, ^34^, ^35^ ^]^ Additionally, since these materials are usually categorized as soft materials, they provide optimal mechanical stiffness for advanced biological applications involving stem cells.^[^ ^27^, ^36^, ^37^ ^]^ Although mechanical strength can be easily modulated via the polymer crosslinking density or techniques such as microfluidic twisting, the as‐synthesized STrIPS‐BIPORES offer intrinsically low stiffness needed for advanced stem cell‐based approaches. These features of interconnected porous biomaterials make them highly conducive to angiogenesis and neovascularization, which are key determinants of successful implantation and optimal defect regeneration.^[^ ^38^, ^39^, ^40^, ^41^, ^42^ ^]^

In this study, we reported for the first time the in vitro and in vivo use of a STrIPS‐based PEGDA‐BIPORES fibrous biomaterial, which possesses an unrestricted, highly bicontinuous interconnected porous framework characterized by uniform pore diameter and optimal surface contour. Our findings illustrate that the distinctive structural characteristics of these PEGDA‐BIPORES‐based biomaterials promote cell homing, attachment, infiltration, proliferation, differentiation, and tissue integration without immune reaction. Of particular significance, our study unveils the applicability of this platform for the culture and maintenance of sensitive cell types, obviating the necessity for specialized basement membrane matrix protein coatings. Therefore, these findings suggest that the unique structural features of the BIPORES scaffold, specifically its bicontinuous interconnected porosity combined with negative Gaussian curvature, are instrumental in facilitating organ‐scale tissue engineering and regeneration. The scaffold's porosity allows for efficient nutrient and cellular transport, fostering an environment that supports cell growth, proliferation, and organ‐scale tissue development. These characteristics make the BIPORES scaffold a promising platform for developing advanced biomaterials that could transform tissue engineering and organ repair.

## 2 Results and Discussion

### 2.1 Fabrication of Polyethylene Glycol Diacrylate (PEGDA)‐BIPORES Fibers

**Figure 1:**
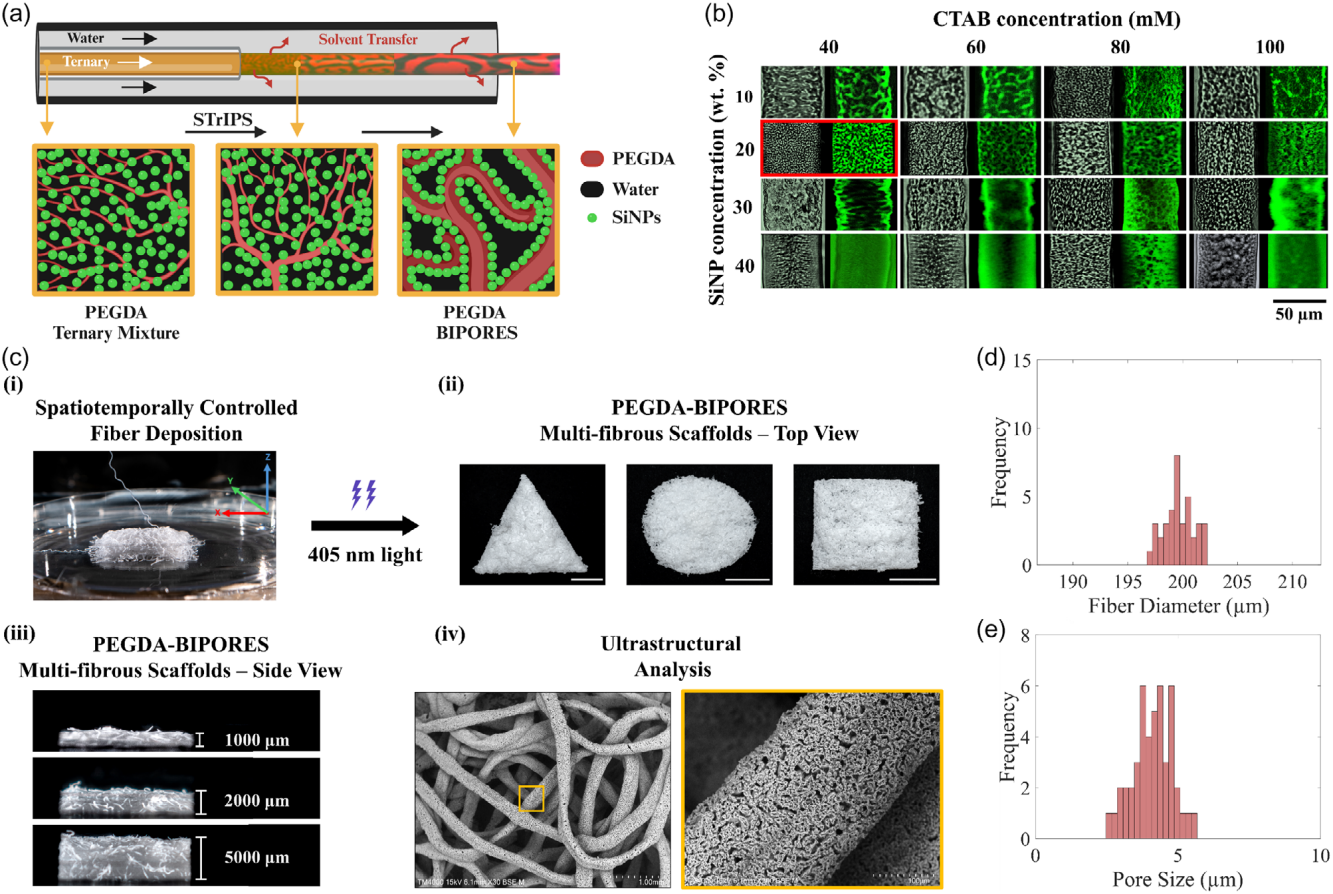
a) Illustration of the BIPORES structure development during STrIPS, showing solvent transfer induces the separation of PEGDA and water phases in the outer capillary, followed by stabilization of the bicontinuous structure by interfacial jamming of the CTAB‐functionalized silica nanoparticles. Black color represents water, red color represents PEGDA, and green color represents silica nanoparticles. **Optimization of the ternary liquid mixture composition**. b) Micrographs of the BIPORES fibers at various concentrations of CTAB (horizontal) in the solvent phase and nanoparticles (vertical) in the water phase. For each concentration, left and right micrographs depict brightfield microscopy and confocal microscopy images, where black color represents the water phase (scale bar: 50 μm). The red box denotes the fabrication condition of 20 wt% silica nanoparticle in the water phase and 40 mM CTAB in the solvent phase, which produced PEGDA‐BIPORES fibers suitable for biological applications and hence used for all subsequent experimentation. **Fabrication of PEGDA‐BIPORES multi‐fibrous 3D meshwork.** c) **(i)** Spatiotemporally controlled advanced deposition of STrIPS‐BIPORES fibers using a custom‐built setup to obtain multi‐fibrous PEGDA‐BIPORES in different **(ii)** geometric configurations (scale bar: 1000 μm) and **(iii)** heights. **(iv)** Scanning electron micrographs showing porous meshwork arrangement of fabricated multi‐fibrous scaffolds along with the characteristic bicontinuous interconnected porous ultrastructure. Distribution of d) fiber diameter and e) pore size of the PEGDA‐BIPORES fibers fabricated at the highlighted (red box in b) optimal concentrations of silica nanoparticles and CTAB.

PEGDA‐BIPORES biomaterials were synthesized using the STrIPS process, which involves flowing a homogeneous ternary liquid blend of hydrophobic PEGDA, water (containing 42 nm silica nanoparticles), and a solvent (ethanol) through a glass capillary into a larger capillary containing a 100 mM NaCl solution. The initial composition was optimized to initialize spinodal decomposition through solvent extraction when in contact with the 100 mM NaCl aqueous phase (Table S1 and Figure S1, Supporting Information). Cetyltrimethylammonium bromide (CTAB) was also added to the ternary mixture via the solvent phase, which helps disperse the nanoparticles in the ternary mixture and makes them partially hydrophobic through electrostatic adsorption (Figure S1b, Supporting Information). This CTAB functionalization enables silica nanoparticle interaction with the hydrophobic PEGDA. As ethanol diffuses out into the stream of the aqueous phase, silica nanoparticles, dispersed in the ternary liquid mixture, accumulate at the PEGDA–water interface to kinetically stop the phase partitioning for stabilizing interwoven bicontinuous architecture (**Figure** **1**a). The stabilization of the phase separation and simultaneous interfacial nanoparticle jamming gives rise to the signature porosity of BIPORES. By adjusting the flow velocity of the ternary liquid and the saline water phase, the PEGDA‐BIPORES was continuously fabricated in the form of cylindrical fibers (Figure S1h–k, Supporting Information). A digital movie of the fiber fabrication process is included in Supporting Information **(Movie S1)**. The lithium phenyl‐2,4,6‐trimethylbenzoylphosphinate (LAP) in the ternary mixture facilitates photocrosslinking of the collected PEGDA‐BIPORES fibers with a 405 nm visible light (Figure S1l, Supporting Information). Successful photocrosslinking of PEGDA‐BIPORES fibers was confirmed by the disappearance of the C=C peak at the wavelength of about 1640 cm^−1^ in the attenuated total reflectance–Fourier‐transform infrared (ATR–FTIR) spectra of the fibers compared to uncrosslinked PEGDA (Figure S1m, Supporting Information). Further details of the BIPORES fabrication procedure are included in Supporting Information (Figure S1‐4, Table S1, Movies S1‐2).

### 2.2 Optimization of PEGDA‐BIPORES for Biological Applications

PEGDA‐BIPORES fibers fabricated at various silica nanoparticle (10, 20, 30, 40 wt% in the aqueous phase) and CTAB (40, 60, 80, 100 mM in the solvent phase) concentrations are shown in Figure 1b. These micrographs demonstrate the interwoven bicontinuous architecture of PEGDA (gray or green color) and water phase (black color). We observed that the domain dimensions, and hence the pore size and the porosity, of the synthesized fibers can be controlled by varying nanoparticle and CTAB concentration in the ternary mixture. Higher nanoparticle concentrations with adequate CTAB resulted in smaller domain sizes due to interfacial jamming in the early stages, while low nanoparticle concentrations with insufficient CTAB resulted in larger domain sizes. Therefore, the domain size of BIPORES fibers decreases with an increase in both the concentration of silica nanoparticles and CTAB. A balance between the concentration of CTAB, which is a cytotoxic agent, and the desirable pore characteristics has to be achieved to ensure good biological compatibility. We considered fibers fabricated with 20 wt% silica nanoparticle and 40 mM CTAB as suitable for cell culture applications. The PEGDA‐BIPORES fibers produced at the selected concentrations require the least quantity of CTAB and have a suitable domain size for biological applications.

A meshwork‐like arrangement of the highly interconnected porous PEGDA‐BIPORES can lead to the development of a cell culture system that allows enhanced diffusion of oxygen and nutrition, critical for full‐thickness cellular survival, functionality, and ultimately integration. As such, we utilized a custom‐built motorized setup to precisely control the deposition of PEGDA‐BIPORES fibers, formed through STrIPS in the microfluidic device, as already mentioned (Figure 1c **(i)**). A video of the advanced controlled deposition of PEGDA‐BIPORES fibers to form a multi‐fibrous scaffold is available in Supporting Information (**Movie S3**). This advanced spatiotemporally controlled continuous deposition of PEGDA‐BIPORES fibers from the STrIPS microfluidic device allowed for the fabrication of highly interconnected porous PEGDA‐BIPORES multi‐fibrous scaffold in both different shapes and heights (Figure 1c **(ii)‐(iii)**). Scanning electron microscope (SEM) micrographs revealed a 3D meshwork architecture with the characteristic BIPORES morphology at the single‐fiber level (Figure 1c **(iv)**). This engineered multi‐scale porosity facilitates enhanced diffusional parameters, thereby positioning the BIPORES meshwork for optimal biological outcomes. More details of the fabrication and post‐processing of the PEGDA‐BIPORES meshwork scaffolds are shown in the schematic (Figure S5, Supporting Information). Additional SEM micrographs of the polymerized PEGDA‐BIPORES fibers, highlighting the hierarchical bicontinuous interconnected porous morphology, are included (Figure S6a–b, Supporting Information). A fiber diameter of about 200 μm and a pore size of about 4‐5 μm were obtained for the PEGDA‐BIPORES fibers produced under the optimal concentrations of 20 wt% silica nanoparticles and 40 mM CTAB (Figure 1d–e).

### 2.3 Material Characterization of PEGDA‐BIPORES

#### 2.3.1 Measurement of Contact Angle of PEGDA‐BIPORES

Surface wettability or hydrophilicity dictates important aspects of biomaterial–biological system interactions like the adhesion of proteins and/or macromolecules, which in turn influences cellular interactions. The surface of the PEGDA‐BIPORES fibers was characterized by measuring the sessile‐drop contact angle (Figure S6c, Supporting Information). Contact angle has been shown to be highly affected by the porosity of a material, with higher porosity leading to a lower contact angle due to a combination of absorption‐related factors such as rate of liquid penetration, droplet size, residence time, etc.^[^ ^43^ ^]^ Thus, a zero contact angle formed by a water droplet in the case of PEGDA‐BIPORES fibers was due to their highly porous and hydrophilic nature in contrast to a contact angle of 40° with the same volume of a water droplet in the case of PEGDA hydrogels indicating their good wettability, adhesiveness, and high surface energy (Figure S6d, Supporting Information). The STrIPS processing of PEGDA polymers results in their highly hydrophilic porous architectures. Hydrophilic biomaterials promote protein adsorption and hence enhance cell adhesion.^[^ ^44^, ^45^ ^]^ As a result, the hydrophilicity of the PEGDA‐BIPORES fibers makes them attractive platforms for biological applications.

#### 2.3.2 Comprehensive Surface Profiling of PEGDA‐BIPORES

**Figure 2:**
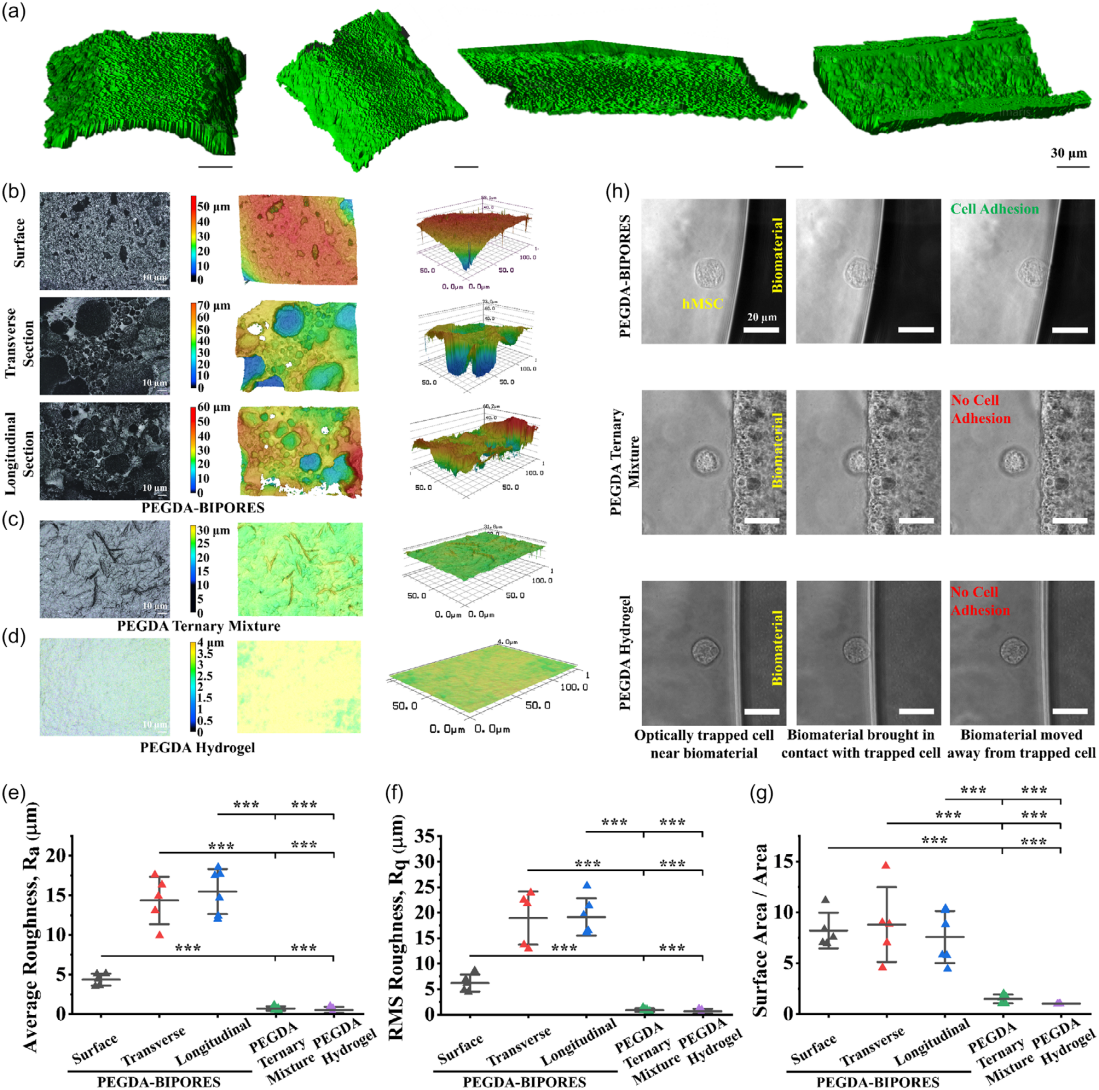
**Evaluation of the surface characteristics of PEGDA‐BIPORES.** a) 3D reconstruction of PEGDA‐BIPORES fiber surface from 2D confocal laser scanning microscopic z‐stack imaging showing feature‐rich fiber surface morphology**. Comparative surface profiling of PEGDA‐BIPORES fibers, PEGDA ternary mixture, and PEGDA hydrogel.** Optical and 3D surface profiles of b) PEGDA‐BIPORES fiber surface, transverse sectional surface, and longitudinal sectional surface, c) PEGDA ternary mixture, and d) PEGDA hydrogel (scale bar: 10 μm). Quantification of e) average surface roughness *R*_a_, f) root mean square surface roughness *R*_q_, and g) surface area‐to‐area ratio from the 3D surface profiles of PEGDA‐BIPORES fibers (top, transverse sectional, and longitudinal sectional surfaces), PEGDA ternary mixture, and PEGDA hydrogel. Data are means ± SD. *p‐*values were determined by Student's *t*‐test (*n* = 4 to 7, ***p < 0.001). **Real‐time assessment of single cell–biomaterial interactions**. h) Microscopic images of PEGDA‐BIPORES fibers, crosslinked PEGDA ternary mixture, and crosslinked PEGDA hydrogel—near, brought in contact with, and pulled away from optically trapped hMSCs (scale bar: 20 μm).

In addition to porosity, surface topographical features of a biomaterial are critical to ensure optimal tissue engineering and regenerative medicine outcomes.^[^ ^46^, ^47^, ^48^, ^49^ ^]^ Surface topography of biomaterials can guide cell behavior and has been leveraged to enhance the bioactivity of scaffolds, the biocompatibility of implantable devices, and regulate host immune response. 3D reconstruction of the PEGDA‐BIPORES fiber surface from 2D confocal z‐stacks revealed a feature‐rich surface morphology (**Figure** **2**a), which prompted a comprehensive surface profiling of the PEGDA‐BIPORES fibers (Figure 2b–g). The PEGDA‐BIPORES surface showed a topography characterized by peaks and valleys, while the PEGDA ternary mixture or PEGDA hydrogel showed uniform surfaces with little to no features. As expected, the PEGDA‐BIPORES fiber sectional surface profiling demonstrated similar hierarchical porous architectures for both the transverse and longitudinal planes, further confirming the interconnectedness of the porosity (Figure 2b–d). Finally, surface roughness measurements of PEGDA‐BIPORES fibers revealed an average surface roughness, *R*_a_ of 4.35 ± 0.75 μm for the top surface, 14.36 ± 3 μm for the transverse sectional surface, and 15.47 ± 2.84 μm for the longitudinal sectional surface. The root mean square surface roughnesses, *R*_q,_ for PEGDA‐BIPORES top surface, transverse sectional surface, and longitudinal sectional surface were 6.18 ± 1.68 μm, 18.95 ± 5.23 μm, and 19.16 ± 3.67 μm, respectively. The surface area‐to‐area ratio for fiber top, transverse sectional, and longitudinal sectional surfaces was 8.21 ± 1.75, 8.8 ± 3.68, and 7.58 ± 2.55, respectively. These surface roughness and specific surface area values were found to be significantly higher than the values obtained for PEGDA ternary mixture (*R*_a_: 0.71 ± 0.27 μm, *R*_q_: 0.99 ± 0.29 μm, and surface area/area: 1.49 ± 0.43) as well as PEGDA hydrogel (*R*_a_: 0.53 ± 0.4 μm, *R*_q_: 0.65 ± 0.46 μm, and surface area/area: 1.02 ± 0.01) (Figure 2e–g). It is well known that surface roughness and specific surface area of a biomaterial strongly govern its interaction with cells, with rougher surfaces shown to promote cellular functions such as adhesion, migration, proliferation, and, most importantly, differentiation.^[^ ^50^, ^51^, ^52^, ^53^ ^]^ Biomaterials with surface unevenness facilitate the anchoring of cells via focal adhesion kinases and ultimately allow enhanced migration on or into the surface in the case of porous substrates. Rougher surfaces also exhibit increased surface area that further boosts cell attachment, migration, and proliferation, steps critical to realizing advanced cellular functions such as differentiation on a biomaterial.

#### 2.3.3 Assessment of Degradation Rate of PEGDA‐BIPORES

The degradation studies (Figure S6e, Supporting Information) conducted on the PEGDA‐BIPORES fibers by incubating them in 1× phosphate‐buffered saline (PBS) under cell culture conditions revealed about 10% weight loss after 3 months, which indicates sufficient stability of these fibers for long‐term use.

### 2.4 Real‐Time Assessment of Single Live Cell–Biomaterial Interactions

Since topographical patterning of biomaterials has profound effects on cell attachment, a crucial step dictating the overall cell–biomaterial compatibility, it is important to investigate cell attachment to a scaffold in real time. As such, we utilized an optical laser tweezers system to directly bring biomaterials in contact with single cells to visualize the cell–biomaterial affinity in real time. The laser tweezer system was used to form an optical trap for live cells at the stage of an inverted microscope (Figure S7, Supporting Information). Optically trapped human mesenchymal stem cells (hMSCs) were tested for their affinity toward the PEGDA‐BIPORES fibers, crosslinked PEGDA ternary mixture, and crosslinked PEGDA hydrogel in two different configurations, tapping mode and contact mode. No attachment between the optically trapped hMSCs and PEGDA‐BIPORES fibers (**Movie S4**, Supporting Information), PEGDA ternary mixture (**Movie S5**, Supporting Information), and PEGDA hydrogel (**Movie S6**, Supporting Information) was observed in the tapping mode configuration. Trapped hMSCs did not adhere to any of the three biomaterials and remained in the optical trap upon the biomaterial being pulled away from the cell. However, contact mode configuration revealed a strong interaction of hMSCs with PEGDA‐BIPORES (**Movie S7**, Supporting Information). When the PEGDA‐BIPORES fiber was allowed to interact with a trapped hMSC for 5 s, the cell adhered to the material and was pulled out of the optical trap upon displacement of the biomaterial away from the trap. The hMSCs did not adhere and remained in the trap even after 10 s of interaction with the PEGDA ternary mixture and PEGDA hydrogel biomaterials (Figure 2h). Furthermore, the hMSCs did not adhere to the PEGDA ternary mixture and PEGDA hydrogel biomaterials even after 10 s of interaction (**Movies S8** and **S9**, Supporting Information). These observations strongly illustrate the higher affinity of hMSCs toward PEGDA‐BIPORES compared to PEGDA ternary mixture or PEGDA hydrogels. We attribute the results observed in the tapping mode configuration to the short interaction time between the biomaterials and the trapped hMSCs. No attachment was observed between hMSCs and any of the biomaterials within 1 s of interaction in contact mode configuration, reiterating the effect of low interaction time on cell adhesion. Similar results were obtained when the experiment was conducted with human induced pluripotent stem cell (hiPSC)‐derived cardiac fibroblasts (hiPSC‐CFs), with cells adhering to the PEGDA‐BIPORES fibers within 10 s of contact, while no cell attachment was observed for PEGDA ternary mixture or PEGDA hydrogel samples (Figure S8, Supporting Information). Videos of the live‐trapped hiPSC‐CF–biomaterial interactions under tapping and contact modes are included in Supporting Information, **Movies S10–15**. The minimum force of adhesion (*F*_*a*dh_) between hMSCs and PEGDA‐BIPORES and hiPSC‐CFs and PEGDA‐BIPORES was estimated using Equation (2) (Experimental Section). The radii of hMSC and hiPSC‐CF were 8.15 μm and 7.05 μm, respectively (Figure 2h and Figure S8, Supporting Information). The estimated force of adhesion (*F*_adh_) for hMSC‐BIPORES interaction was 2.606 nN, and for hiPSC‐CF–BIPORES interaction was 2.255 nN.

### 2.5 Cytocompatibility of the PEGDA‐BIPORES Fibers

#### 2.5.1 Assessment of Cytocompatibility of PEGDA‐BIPORES Fibers with hMSCs and C2C12 Cells

Experimentally, the STrIPS‐based BIPORES morphology was found to be crucial for transforming a bioinert PEGDA (M_*n*_ 250) hydrogel to a bioactive platform, as hMSCs cultured on the polymerized ternary liquid without STrIPS processing showed no cell adhesion/survival at days 3 and 7 (Figure S9, Supporting Information). Cell studies with different cell types were conducted on the PEGDA‐BIPORES fibers to extensively characterize cell behavior in these systems. The viability of hMSCs growing on the PEGDA‐BIPORES fibers was assayed over 14 days. The fluorescent microscopic images from the live/dead assays performed on days 1, 4, 7, and 14 post‐seeding of hMSCs are shown in Figure S10a–c, Supporting Information, where green fluorescence indicated live cells and red fluorescence marked dead cells. The results show a progressive increase in live cells and a negligible proportion of dead cells on the PEGDA‐BIPORES fibers, with a 99% cell viability observed on day 14 (Figure S10c, Supporting Information). Phalloidin/4′,6‐diamidino‐2‐phenylindole (DAPI) fluorescent staining and imaging were conducted to study the morphology of the attached cells in terms of actin cytoskeletal spreading on the fibers. The phalloidin/DAPI staining data on days 1, 4, 7, and 14 after seeding of hMSCs showed a progressive increase in cell proliferation as determined by an increasing number of cell nuclei (blue) with a remarkable increase in the F‐actin cytoskeleton (green) (Figure S10b–d, Supporting Information). To better understand the metabolic health of the stem cells on and around the PEGDA‐BIPORES fibers, the PrestoBlue cell viability assay was performed, which showed a dramatic increase in metabolically intact cells over the span of the cultures (Figure S10e, Supporting Information). Since both the positive controls and the PEGDA‐BIPORES fibers exhibited a statistically significant increase in metabolically active cells over the culture duration, the PEGDA‐BIPORES fibers can be considered to be highly cytocompatible. Similar results were obtained when the in vitro cytocompatibility studies of the PEGDA‐BIPORES fibers were performed with immortal mouse myoblast C2C12 cells (Figure S11, Supporting Information). These findings highlight the importance of the BIPORES morphological modification in imparting biological activity to an otherwise inert polymeric biomaterial. The enhanced surface topographical features, increased surface area, and highly interconnected porosity specifically contribute to the extensive cellular performance of the PEGDA‐BIPORES biomaterial. Thus, STrIPS processing can be utilized as a novel method to convert suitable bioinert materials into biocompatible bioactive BIPORES materials.

#### 2.5.2 Osteogenic Differentiation of hMSCs on PEGDA‐BIPORES Fibers

**Figure 3:**
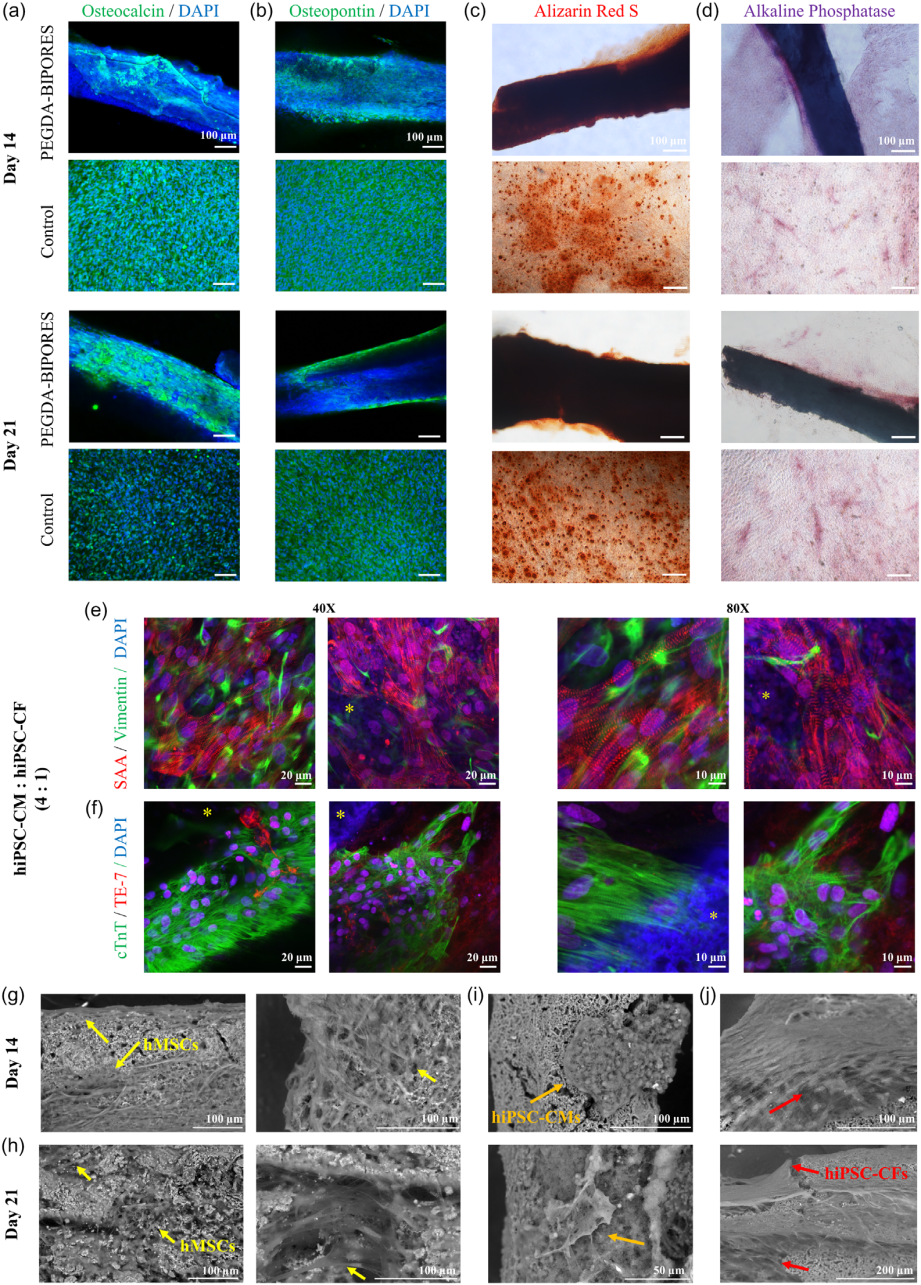
**Osteogenic differentiation of hMSCs on PEGDA‐BIPORES fibers.** a,b) Immunofluorescence staining and confocal microscopic imaging of hMSCs cultured on PEGDA‐BIPORES fibers and tissue culture plates (positive control) for 14 and 21 days. Detection of osteogenic differentiation by a) osteocalcin (OCN) and b) osteopontin (OPN), with the nuclei counterstained by DAPI. c,d) Cytochemical analysis of osteogenic differentiation of hMSCs cultured on PEGDA‐BIPORES fibers and tissue culture plates. Calcium deposition and alkaline phosphatase activity during osteogenic differentiation, demonstrated by c) alizarin red S (ARS) and d) alkaline phosphatase (ALP) stains, respectively (scale bar: 100 μm). **Co‐culture of hiPSC‐CMs and hiPSC‐CFs on PEGDA‐BIPORES fibers.** e,f) Immunofluorescence co‐staining and confocal microscopic imaging of co‐cultured hiPSC‐CMs and hiPSC‐CFs (4:1) seeded on PEGDA‐BIPORES fibers. Beating hiPSC‐CMs and hiPSC‐CFs on the fibers co‐immunostained for e) sarcomeric α‐actinin (SAA, red, hiPSC‐CMs) and vimentin (green, hiPSC‐CFs) and f) cardiac troponin T (cTnT, green, hiPSC‐CMs) and anti‐fibroblast TE‐7 (red, hiPSC‐CFs), with the nuclei counterstained by DAPI, on day 12 post‐seeding. The yellow asterisk represents the BIPORES biomaterial. **Morphological assessment of cell‐laden PEGDA‐BIPORES fibers.** g,h) Scanning electron microscope (SEM) micrographs of hMSCs (yellow arrow) grown and osteogenically differentiated on PEGDA‐BIPORES fibers for g) 14 and h) 21 days at different magnifications. i,j) SEM micrographs of i) hiPSC‐CMs (orange arrow), and j) hiPSC‐CFs (red arrow), cultured on PEGDA‐BIPORES fibers, at different magnifications.

The ability of a biomaterial to support critical cellular functions, such as differentiation, is an important indicator of its biofunctionality. To evaluate the osteoconductive potential of PEGDA‐BIPORES fibers, an osteogenic differentiation study was performed. As shown in **Figure** **3**, osteogenic differentiation of hMSCs on PEGDA‐BIPORES fibers was assessed by immunostaining for osteogenic markers such as osteocalcin (OCN) and osteopontin (OPN) proteins, followed by confocal laser scanning microscopic imaging (Figure 3a–b) in addition to staining for calcium mineral deposits (alizarin red S (ARS)) and alkaline phosphatase (ALP) activity and brightfield microscopic imaging (Figure 3c–d) on days 14 and 21. The hMSCs cultured on PEGDA‐BIPORES fibers showed intense immunofluorescence on both day 14 and day 21, confirming their differentiation to osteoblasts and bone‐specific cells such as osteoclasts (Figure 3a–b). The ARS and ALP staining on both day 14 and day 21 time points reinforced the successful osteogenic differentiation of the hMSCs (Figure 3c–d). The presence of silica nanoparticles in the PEGDA‐BIPORES fibers likely enhanced the osteogenic differentiation of hMSCs due to the well‐established osteoconductive properties of silica.^[^ ^35^, ^54^, ^55^, ^56^ ^]^ Interestingly, the PEGDA‐BIPORES fibers also demonstrated osteoinductive properties (Figure S12, Supporting Information) as hMSCs cultured on the fibers in the absence of osteogenic differentiation induction medium showed immunofluorescence for osteogenic differentiation markers such as OCN and OPN. hMSCs cultured in the absence of PEGDA‐BIPORES fibers did not differentiate into osteoblasts. These results further reinforce the osteoinductive properties of the PEGDA‐BIPORES fibers, which are likely imparted by the silica nanoparticles residing at the bicontinuous interface.

#### 2.5.3 hiPSC‐derived Cardiac Cell Culture on PEGDA‐BIPORES Fibers

WTC‐11 hiPSCs were differentiated into cardiomyocytes (hiPSC‐CMs) through an established protocol as depicted in Figure S13a, Supporting Information. Synchronically contracting hiPSC‐CMs, typically around days 10–12 of differentiation, indicated the readiness of the CMs for seeding on PEGDA‐BIPORES fibers. The hiPSC‐CM‐laden PEGDA‐BIPORES fibers were subsequently maintained until synchronized contractions of the hiPSC‐CMs became evident, typically around days 3–5 post‐seeding (digital movie of beating hiPSC‐CMs on PEGDA‐BIPORES fibers shown in **Movies S16** and **S17**, Supporting Information), following which the samples were retrieved for analysis (Figure S13b‐e, Supporting Information). Evaluation of cell viability was performed using a calcein‐AM/ethidium homodimer‐I‐based live/dead assay (Figure S13b, Supporting Information). The results highlighted robust hiPSC‐CM viability on and around the PEGDA‐BIPORES fibers, with negligible cell death. F‐actin cytoskeletal spreading of the hiPSC‐CMs on the PEGDA‐BIPORES fibers was assessed using phalloidin/DAPI fluorescent staining (Figure S13c, Supporting Information). The representative images revealed the actin cytoskeletal morphologies of the hiPSC‐CMs attached to PEGDA‐BIPORES fibers. Additionally, the cell‐laden PEGDA‐BIPORES samples were subjected to immunofluorescent staining targeting CM biomarkers such as sarcomeric α‐actinin (SAA) and cardiac troponin T (cTnT) (Figure S13d–e, Supporting Information). The data underscored the significant expression of both proteins within the beating hiPSC‐CM structures on the PEGDA‐BIPORES fibers. Similarly, hiPSC‐CFs were differentiated from WTC‐11 hiPSCs using a protocol as depicted in Figure S13f, Supporting Information. hiPSC‐CFs at passage number 5 were seeded on the PEGDA‐BIPORES fibers and cultured for 14 days, resulting in extensive cell proliferation and attainment of confluency on the fibers as observed from brightfield microscopic evaluation of cell viability and morphology (Figure S13g, Supporting Information). Phalloidin/DAPI staining (Figure S13h, Supporting Information) and immunofluorescence staining for CF markers such as TE‐7 and vimentin (Figure S13i–j, Supporting Information) confirmed successful maintenance of hiPSC‐CFs on the PEGDA‐BIPORES fibers. These results taken together indicate the immense potential of PEGDA‐BIPORES fibers as a next‐generation scaffold for cardiac tissue engineering.

**Figure 4:**
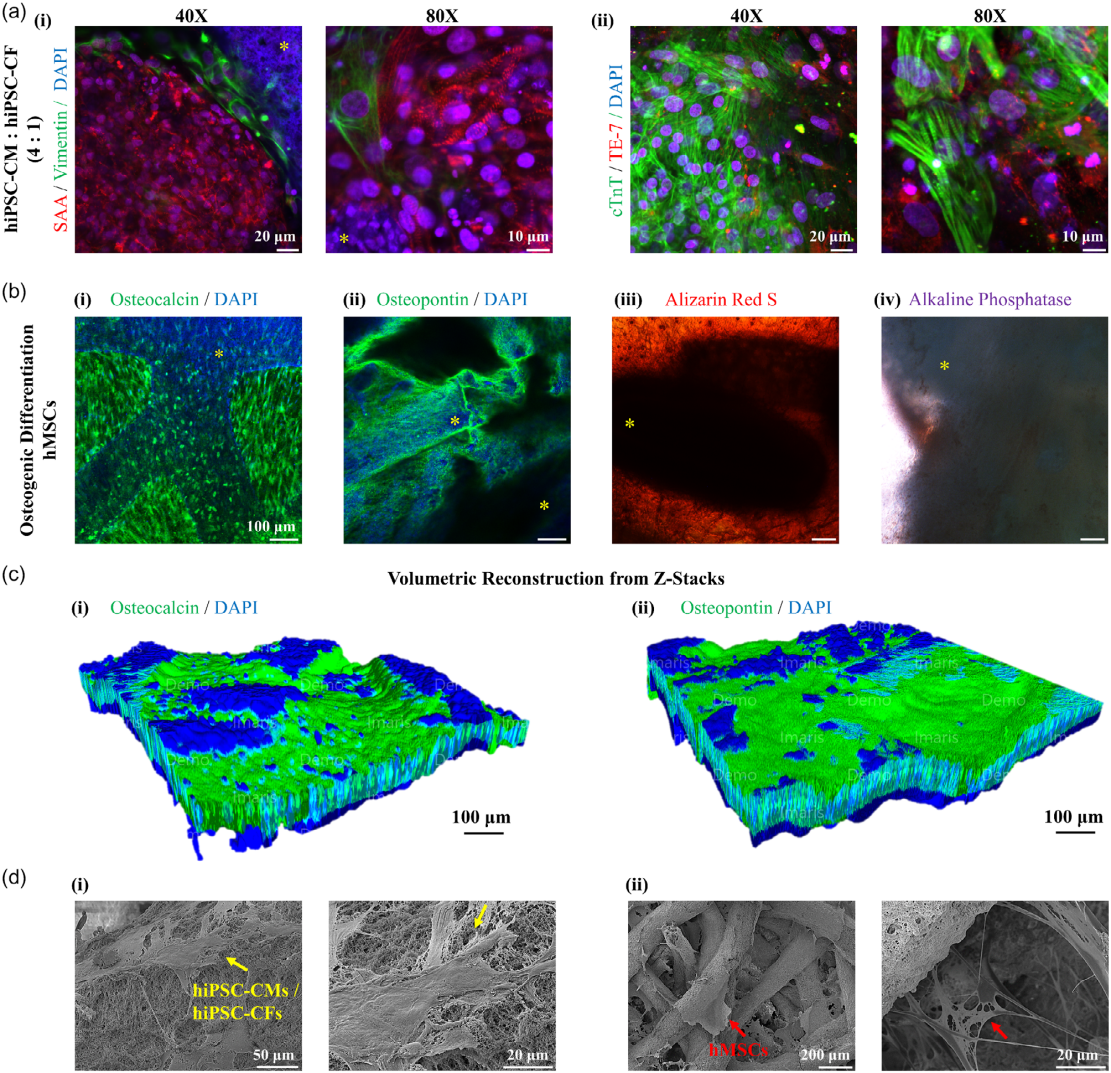
**Bioactivity of hiPSC‐CMs and hiPSC‐CFs co‐cultured on PEGDA‐BIPORES multi‐fibrous scaffolds**. a) Immunofluorescence co‐staining and confocal microscopic imaging of co‐cultured hiPSC‐CMs and hiPSC‐CFs (4:1) seeded on PEGDA‐BIPORES 3D meshwork scaffold. Beating hiPSC‐CMs and hiPSC‐CFs on the multi‐fibrous scaffold co‐immunostained for **(i)** sarcomeric α‐actinin (SAA, red, hiPSC‐CMs) and vimentin (green, hiPSC‐CFs), and **(ii)** cardiac troponin T (cTnT, green, hiPSC‐CMs) and anti‐fibroblast TE‐7 (red, hiPSC‐CFs), with the nuclei counterstained by DAPI, on day 12 post‐seeding. The yellow asterisk represents the biomaterial. **Osteogenic differentiation of hMSCs on PEGDA‐BIPORES multi‐fibrous scaffolds.** b) **(i, ii)** Immunofluorescence staining and confocal microscopic imaging of hMSCs cultured on PEGDA‐BIPORES multifibrous meshwork for 21 days. Detection of osteogenic differentiation by **(i)** osteocalcin (OCN) and **(ii)** osteopontin (OPN), with the nuclei counterstained by DAPI (scale bar: 100 μm). **(iii, iv)** Cytochemical analysis of osteogenic differentiation of hMSCs cultured on PEGDA‐BIPORES meshwork. Determination of calcium deposition and alkaline phosphatase activity during osteogenic differentiation by **(iii)** alizarin red S (ARS) and **(iv)** alkaline phosphatase (ALP) assays, respectively (scale bar: 100 μm). The yellow asterisk represents the biomaterial. **3D volumetric reconstruction of cell‐laden PEGDA‐BIPORES multi‐fibrous scaffolds**. c) Volumetric rendering of cell‐laden PEGDA‐BIPORES fibrous meshwork using confocal microscopic z‐stack images of **(i)** OCN/DAPI–and **(ii)** OPN/DAPI‐stained samples. **Morphological assessment of cell‐laden PEGDA‐BIPORES multi‐fibrous scaffolds**. d) Scanning electron microscope (SEM) micrographs of **(i)** co‐cultured hiPSC‐CMs/ hiPSC‐CFs (yellow arrow) and **(ii)** hMSCs (red arrow) grown and osteogenically differentiated on PEGDA‐BIPORES fibrous meshwork for 21 days, at different magnifications.

Based on the successful cardiac cell viability and functionality under mono‐culture conditions, co‐culture of hiPSC‐CMs and hiPSC‐CFs was done on the PEGDA‐BIPORES fibers at a ratio of 4:1, which was chosen to mimic proportions found in healthy hearts according to existing literature.^[^ ^57^, ^58^ ^]^ Co‐culture of hiPSC‐CMs and hiPSC‐CFs better recapitulates the physiological composition of the cardiac tissue. Characteristic beating phenotype of the hiPSC‐CMs, co‐cultured with hiPSC‐CFs, on the PEGDA‐BIPORES fibers, was observed as early as the first day after cell seeding (**Movies S18–19**, Supporting Information). The spontaneous beating rate of the hiPSC‐CMs increased from 9.8 ± 1.6 beats per minute on day 6 post‐seeding to 16 ± 3.9 beats per minute on day 12 post‐seeding (Figure S14, Supporting Information). Co‐immunostaining the co‐cultured hiPSC‐CMs/hiPSC‐CFs for cardiac biomarkers, on day 12 post‐seeding, enabled the simultaneous visualization of distinct cellular structural components (Figure 3e–f). First, immunocytochemical co‐staining for SAA and vimentin was done to characterize the cell distribution on the PEGDA‐BIPORES fibers. The characteristic SAA striation bands were present in all the SAA‐stained samples, with the SAA bands/Z discs running across PEGDA‐BIPORES fibers (Figure 3e). The average sarcomere length (the average Z disc to Z disc distance) was quantified to be about 1.35 ± 0.14 μm (Figure S15, Supporting Information). Similarly, the expression of vimentin on PEGDA‐BIPORES fibers confirmed the presence of hiPSC‐CFs. Further validation of hiPSC‐CM and hiPSC‐CF phenotypes was achieved through the co‐immunostaining of the samples for cardiac troponin T (cTnT) and fibroblast TE‐7, respectively (**Figure** **4**f). The immunostaining results demonstrated strong expression of cTnT with well‐defined and prominent sarcomeric organization in hiPSC‐CMs. The presence of hiPSC‐CFs on the PEGDA‐BIPORES fibers was confirmed via positive fibroblast TE‐7 stain. The strong expressions of the cardiac biomarkers in the co‐cultured cardiac cells atop the PEGDA‐BIPORES reiterate the potential of this synthetic platform for cardiac tissue engineering and regeneration. These findings are remarkably interesting, since to the best of our knowledge, we are the first group to show superior cell viability and characteristic beating functionality of hiPSC‐CMs/hiPSC‐CFs in a completely artificial/synthetic setting without basement membrane matrix protein‐coated surfaces or any additional protein sources other than the cell maintenance media. Owing to the inherent sensitivity and susceptibility of hiPSC‐CMs/hiPSC‐CFs to culture conditions, our findings demonstrate that the application of PEGDA‐BIPORES fibers as a cell culture substrate surpasses conventional approaches, hinting at the potential utility of this innovative platform for pivotal regenerative medicine/tissue engineering applications.

#### 2.5.4 SEM Analysis of Cell‐laden PEGDA‐BIPORES Fibers

After 14 and 21 days of osteogenic differentiation of the hMSCs, the surface of the PEGDA‐BIPORES fibers was covered by a dense cellular layer, and cells migrated significantly inside the scaffold as revealed by cross‐sectional SEM imaging (Figure 3g–h). The heightened surface‐to‐volume ratio of the porous fibers allowed hMSCs to attach, grow, and spread on the scaffold. The flattened cell structure, coupled with remarkable extension both within and surrounding the interconnected porous fibers, pointed to robust cell attachment and growth. Further, as discussed already, silica nanoparticle‐laden scaffolds have inherent osteoinductive and osteoconductive properties. Thus, the silica nanoparticles in the 3D porous PEGDA‐BIPORES fibers likely upregulated the hMSC osteogenic differentiation. Similarly, the attachment of hiPSC‐CMs and hiPSC‐CFs seeded on PEGDA‐BIPORES fibers is shown in Figure 3i,j, respectively. The hiPSC‐CMs exhibited excellent interaction with the fibers, accompanied by typical morphology and contractions, as shown already. hiPSC‐CFs demonstrated great adhesion and preferential migration toward the biomaterial, resulting in the attainment of confluency by day 14 of culture. The hiPSC‐CF ECM pervades throughout the fiber structure as revealed by the SEM micrographs of ruptured fibers.

### 2.6 Assessment of PEGDA‐BIPORES Multi‐fibrous 3D Meshwork

To assess the cytocompatibility of the PEGDA‐BIPORES multi‐fibrous system, hiPSC‐CFs were seeded on the scaffolds and cultured for 14 days. Brightfield microscopic images were acquired to show gross cell activity on the 3D meshwork scaffolds (Figure S16a, Supporting Information). On day 14, the PEGDA‐BIPORES samples were stained with the live/dead and phalloidin/DAPI to assess cell viability and cellular structures, respectively (Figure S16b **(i)‐(ii)**, Supporting Information). Additionally, immunostaining for CF biomarkers such as vimentin and TE‐7 was performed to confirm the maintenance of hiPSC‐CFs within the scaffolds, with DAPI being the nuclear counterstain (Figure S16b **(iii)‐(iv)**, Supporting Information). Depth‐mode high‐magnification SEM imaging further confirmed extensive hiPSC‐CF ECM deposition throughout the volume of the multi‐fibrous structure (Figure S16c, Supporting Information). Prior to confocal microscopic imaging, the stained PEGDA‐BIPORES scaffolds were ruptured in order to investigate cellular activity within the bulk of the scaffolds. The imaging results revealed the extensive survival, proliferation, and integration of the hiPSC‐CFs within the PEGDA‐BIPORES meshwork. For the beating hiPSC‐CMs (**Movie S20**, Supporting Information) and hiPSC‐CFs co‐cultured (4:1 ratio) on PEGDA‐BIPORES 3D meshwork, co‐immunostaining for cardiac biomarkers SAA/vimentin and cTnT/TE‐7 showed prominent Z‐discs and cTnT expression for hiPSC‐CMs, while strong expressions of vimentin and TE‐7 confirmed the successful maintenance and integration of hiPSC‐CFs within the PEGDA‐BIPORES multi‐fibrous scaffolds (Figure 4a **(i)‐(ii)**). In light of the single‐fiber cellular performance, this outcome was expected since the highly interconnected bicontinuous porous nature and the surface characteristics of the PEGDA‐BIPORES are preserved at the multi‐fibrous level.

To further validate the capabilities of the multi‐fibrous PEGDA‐BIPORES, hMSCs were seeded on the scaffolds, pre‐cultured for 2 days in maintenance medium, and then subjected to osteogenic differentiation induction medium for 21 days. Brightfield microscopic imaging was done to observe mineral deposition, a key marker of osteogenic differentiation, on the 3D meshwork scaffolds (Figure S17, Supporting Information). Immunostaining for osteogenic differentiation markers such as OCN and OPN revealed extensive expression of both markers within the scaffolds (Figure 4b **(i)‐(ii)**). These results were further reinforced by successful cytochemical assays for osteogenic differentiation, such as the ARS and the ALP activity (Figure 4b **(iii)‐(iv)**). Taken together, these results substantiate the excellent ability of the PEGDA‐BIPORES multi‐fibrous meshwork platform to support vital cellular functionalities such as differentiation. All immunostained samples were split in half prior to confocal laser scanning microscopic imaging to assess cellular performance at the core of the scaffold. The results hint at the conducive diffusional parameters in the multi‐fibrous PEGDA‐BIPORES, conferred by the specially developed meshwork deposition technique, necessary for sustaining cells throughout the volume of the scaffolds. Furthermore, confocal z‐stack imaging of immunostained cell‐laden samples was used to perform volumetric reconstruction of the cell‐scaffold structures, revealing cellular presence in the bulk of the materials (Figure 4c **(i)‐(ii)**).

To further study the cellular integration within the volume of the PEGDA‐BIPORES meshwork system, high‐magnification SEM imaging of the cell‐laden scaffolds was done in the depth mode (Figure 4d). In the case of both co‐cultured hiPSC‐CMs/hiPSC‐CFs and osteogenically differentiated hMSCs, extensive ECM deposition was found within the volume of the scaffolds, further establishing the extensive cytocompatibility of the multi‐fibrous PEGDA‐BIPORES platform. These results reiterate the synergistic enhancement of bioactivity of the synthetic bioinert PEGDA‐based BIPORES platforms, due to the highly interconnected bicontinuous porosity and the characteristic surface topography.

### 2.7 In vivo Biocompatibility of PEGDA‐BIPORES

Following the promising in vitro cytocompatibility characterization of the PEGDA‐BIPORES fibrous scaffolds, it was imperative to perform in vivo assessments to evaluate their potential for clinical translation. These studies provide critical insights into the scaffolds’ performance in a physiological environment, ultimately determining their suitability for therapeutic applications.

**Figure 5:**
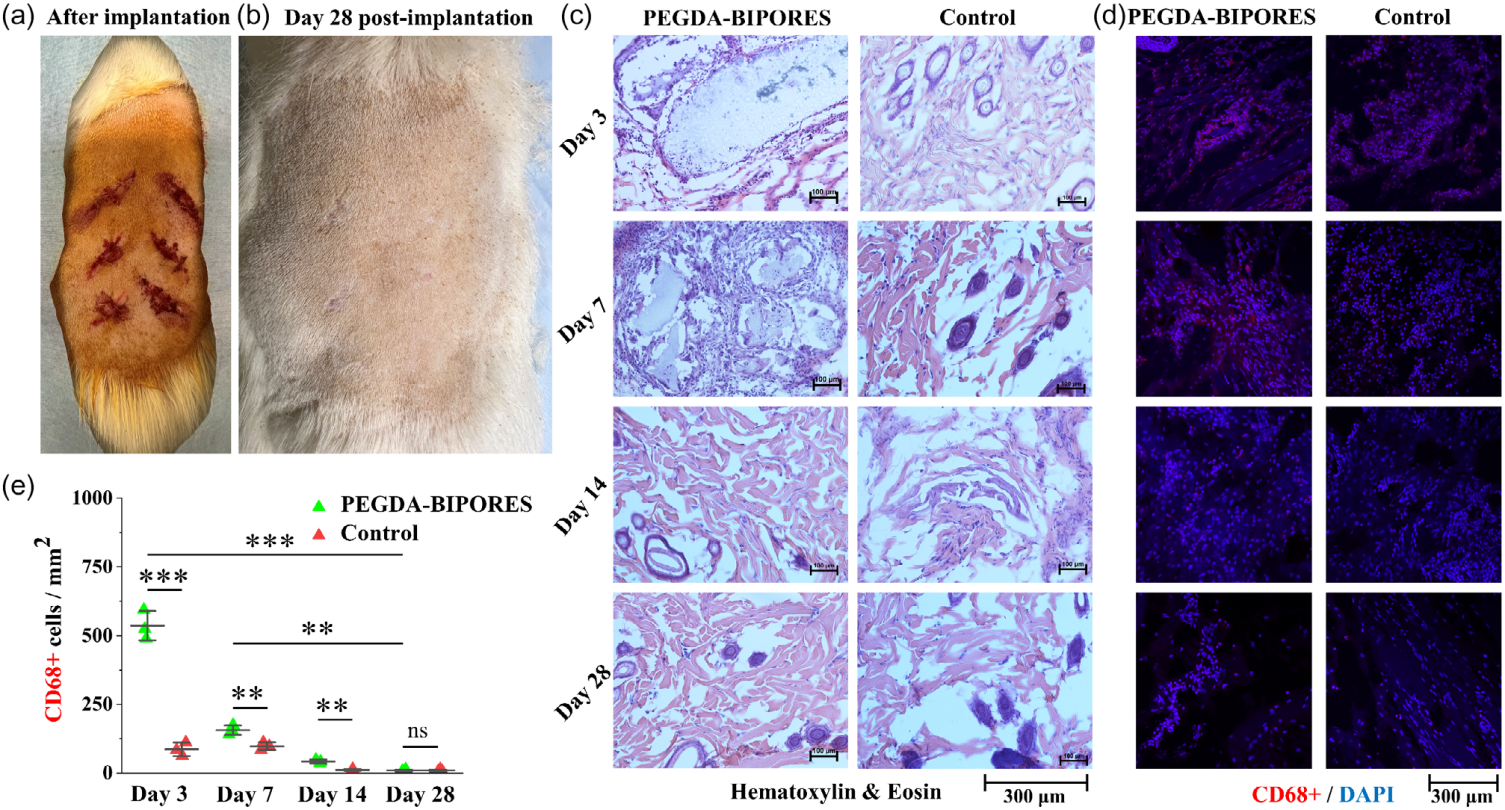
**Subcutaneous implantation of PEGDA‐BIPORES fibers in a rat model.** Photographs, taken on a) the day of the surgical procedure, and b) day 28 post‐surgery, showing complete healing and no signs of toxicity. The two bottom‐right incisions in each animal did not receive any biomaterial and acted as controls. **Histological analysis of skin tissue by H&E staining**. c) Normal histological appearance of the skin tissue was observed in all groups with no distinction between control and experimental groups, indicating biocompatibility of PEGDA‐BIPORES fibers (scale bar: 300 μm). **Immunofluorescence staining of skin tissue for CD68+ macrophages**. d) Fluorescent immunohistochemical staining of subcutaneously implanted PEGDA‐BIPORES biomaterial showing significant macrophage (CD68+ cells) infiltration, initially at day 3, which significantly declined by days 7, 14, and 28, in all groups (scale bar: 300 μm). e) Quantification of inflammatory responses to the PEGDA‐BIPORES fibers based on CD68+ immunofluorescence images. Data are means ± SD. *p*‐values were determined by Student's *t*‐test and one‐way ANOVA with Tukey's post‐hoc test (*n* = 3 (biological replicates), *p < 0.05, **p < 0.01, ***p < 0.001, ns–non‐significant).

Subcutaneous implantation of the PEGDA‐BIPORES fibers into Wistar rats was performed to evaluate their in vivo biocompatibility. Digital photographs of the surgical sites taken right after the implantation procedure and after 28 days showed complete healing of the defects with negligible signs of scarring and difference compared to control defects without the fibers (**Figure** **5**a–b). Implant/skin tissue samples extracted on days 3, 7, 14, and 28 were analyzed to determine the extent of integration of the host tissue with the implanted fibers. Figure 5c–d shows the representative hematoxylin & eosin (H&E) histological and fluorescent immunohistochemical imaging data after 3, 7, 14, and 28 days of implantation, respectively. The morphology of tissue surrounding the implanted fibers was characterized by H&E staining and imaging. Upon histological examination of the explanted samples, it was observed that predominantly non‐inflammatory tissue had grown into the fibers, and there was no significant deposition of fibrous collagenous capsules. Interestingly, the morphology of PEGDA‐BIPORES tissue displayed no significant differences compared to the control tissue, suggesting that the PEGDA‐BIPORES fibers did not induce any adverse inflammatory reactions. This finding was further supported by immunohistofluorescent evaluation of the extracted tissues. To characterize the local immune response, fluorescent immunohistochemical staining targeting CD68+ pan‐macrophages was performed. The implantation of PEGDA‐BIPORES fibers resulted in the invasion of CD68+ macrophages at the boundary between the fibers and subcutaneous tissue, particularly evident on day 3; however, their presence significantly decreased by day 28. Figure 5e demonstrates the reduction in the number of CD68+ cells over time, indicating the absence of inflammation. These results strongly indicate that PEGDA‐BIPORES fibers triggered insignificant inflammatory responses in vivo. The lack of collagenous encapsulation at the implant–tissue interface meant that the fibers were not rejected by the foreign body response.

The tissue response to implanted materials is heavily influenced by the microstructure of the implant.^[^ ^59^ ^]^ The arrangement of pores within the implant can impact cell migration, macrophage polarization, and blood vessel formation. A unique feature of BIPORES biomaterials is their self‐assembly during spinodal decomposition, resulting in interfaces with negative Gaussian curvature and a maze‐like pore network that remains open and unobstructed. This advantageous surface with hyperbolic curvature allows cells to migrate freely within the BIPORES implants. The consistent pore size further facilitates the penetration of large blood vessels deep into the implants, promoting uninhibited infiltration of macrophages and native cells. This open infiltration may significantly delay or prevent the formation of a fibrous envelope, ultimately increasing the longevity of implants. Recent studies exploring the effects of substrate curvature on cell behavior, particularly adhesion, migration, and differentiation, have revealed distinct orientations of cell apical stress fibers on surfaces with different Gaussian curvatures.^[^ ^59^ ^]^ Flat surfaces or cylinders with zero Gaussian curvature induce cytoskeletal microfilaments aligned along the path of cell migration, while surfaces with negative Gaussian or concave curvature cause microfilaments to position orthogonally to the direction of cell movement. Considering the prevalence of saddle‐like concave curvatures on the interior of BIPORES materials, it is reasonable to expect a significant impact on cell–biomaterial interplay inside the BIPORES biomaterial.

## 3 Conclusion

The STrIPS method of making bicontinuous interconnected microporous PEGDA‐BIPORES hydrogels represents a paradigm shift in the design of biomaterials that can convert biocompatible bioinert materials into bioactive substrates. We showed that this new class of bicontinuous biomaterials possesses optimal surface topography and interconnected pore architecture that facilitates cell attachment, proliferation, and enhances osteogenic differentiation of hMSCs. The ability of the completely synthetic PEGDA‐BIPORES biomaterial to support the maintenance and beating of hiPSC‐CMs and hiPSC‐CFs, which are sensitive to culture conditions, without any additional protein coatings, is reported. We established an advanced controlled deposition technique for the fabrication of multi‐fibrous meshwork‐like STrIPS‐based BIPORES scaffolds. The hMSCs or hiPSC‐CMs/hiPSC‐CFs cultured on the meshwork demonstrated successful cell growth, proliferation, osteogenic differentiation of hMSCs, and ultimately integration throughout the bulk of the scaffolds. In vivo studies with rats demonstrated that subcutaneously implanted PEGDA‐BIPORES biomaterials did not induce a sustained inflammatory response and were effectively integrated into connective tissues, confirming their excellent biocompatibility. These findings highlight the potential of the BIPORES approach for developing biomaterials that can enhance the functionality and durability of biomaterials to fit a myriad of tissue engineering and regenerative medicine applications.

## 4 Experimental Section

4.1

4.1.1

Most of the chemicals were procured from Sigma‐Aldrich (St. Louis, MO, USA) unless otherwise stated. We used analytical reagent‐grade chemicals without additional purification. W. R. Grace & Co. supplied the SiO_2_ nanoparticles (Ludox PW‐50, 42 nm), while CTAB and LAP were purchased from Sigma‐Aldrich and used in their received form. Deionized (DI) water and ethanol (200 proof) were utilized for all our experiments. The syringe pumps were purchased from KD Scientific (Holliston, MA, USA), and a 405 nm visible light on the Allevi 2 bioprinter (Allevi, 3D Systems) was used for photocrosslinking.

##### Microfluidic Device Construction

For the production and characterization of PEGDA‐BIPORES fibers, a custom‐made microfluidic device was utilized. Two different shapes of glass capillaries, one round (outer diameter 840 μm, inner diameter 600 μm) and the other square (outer diameter 1500 μm, inner diameter 1050 μm), were custom ordered from VitroCom, NJ. The tip diameter of the round glass capillary was reduced to 200–300 μm using a micropipette puller (Sutter Instrument). By inserting the smaller round capillary into the larger square capillary and aligning them concentrically, the microfluidics‐based device for PEGDA‐BIPORES fiber fabrication was constructed. To prevent undesired adsorption of the fiber‐forming mixture on the walls of the capillaries, the capillaries were coated with polydiallyldimethylammonium chloride (PDADMAC). The capillaries were first flushed with a solution containing PDADMAC (0.5 wt%) and NaCl (0.5 M), followed by thorough rinsing with water. Additional details for making and testing the microfluidic devices are included in the Supporting Information.

##### Precursor Ternary Mixture Preparation

To produce PEGDA‐BIPORES fibers, we employed the following materials: Ludox PW‐50 colloidal silica (SiO_2_, 50 wt% suspension in water), CTAB, PEGDA (M_*n*_ 250, Sigma‐Aldrich), absolute ethanol (analytical grade), LAP, and NaCl. The preparation of the ternary liquid mixture involves 1) aqueous phase–a suspension of Ludox PW‐50 (42 nm SiO_2_ nanoparticles, pH adjusted to 3), LAP, and NaCl in DI water, 2) polymer phase–PEGDA, and 3) solvent phase–a solution of CTAB in pure ethanol. The stock SiO_2_ suspension had a silica concentration of 50 wt% and a pH value between 9 and 10. We adjusted the pH of this suspension to 3 by gradually adding hydrochloric acid. For preparing a 10 mL solution used for generating polymerized PEGDA‐BIPORES fibers with 40 mM CTAB (in the solvent phase) and 20 wt% silica nanoparticles (in the aqueous phase), 40% v/v PEGDA, 30% v/v ethanol, and 30% v/v water phase were mixed. This emulsion corresponds to 4 mL of PEGDA, 3 mL of a 40 mM CTAB solution in pure ethanol, and 3 mL of the aqueous solution (1.2 mL of the 50 wt% Ludox PW‐50 stock, 1.4 mL of a 2.5 wt% LAP solution in 16.79 mM NaCl, and 0.4 mL of 16.79 mM NaCl). A translucent and uniformly mixed solution with evenly distributed silica nanoparticles was obtained by simply shaking the mixture.

##### PEGDA‐BIPORES Fiber Extrusion Process

The production of PEGDA‐BIPORES fibers using the STrIPS method was carried out using the microfluidic device. Specifically, the ternary liquid mixture and a 100 mM NaCl solution in plastic syringes were pumped via polytetrafluoroethylene (PTFE) tubing into the inner and outer capillaries, respectively, using syringe pumps. The capillary device was positioned vertically using finger clamps, allowing the liquids in the two channels to flow in the same direction. The NaCl phase in the outer capillary facilitated the extraction of ethanol from the ternary mixture, triggering phase separation. Additionally, the outer flow assisted in aligning the fibers and ensuring homogeneous transfer of mass. The distance between the outlets of the smaller capillary and the larger capillary can be adjusted as needed. The fabricated PEGDA‐BIPORES fibers were collected by submerging the microfluidic device in a container, such as a glass bowl, filled with DI water. Deposition of fibers on a surface in an aligned fashion was achieved by moving the collection bowl during the collection process. The flow rate for the outer NaCl phase was set to 0.7 mL min^−1^, while the ternary fluid was flown at 5 mL h^−1^. The resulting fibers were subsequently photocrosslinked using a 405 nm light of the Allevi 2 bioprinter, at an intensity of 20 mW/cm^2^, for a duration of 5 min. After rinsing with 1× PBS, the fibers were allowed to air dry overnight and stored in air‐tight, covered containers until further experimentation.

##### Fabrication of PEGDA‐BIPORES Multi‐fibrous Meshwork Scaffolds

Advanced controlled fabrication of PEGDA‐BIPORES multi‐fibrous meshwork‐like scaffolds was achieved via modification of an Allevi 2 bioprinter while utilizing the existing X/Y control gantry. An acrylic water bath was fabricated and attached to the Z stage along with a microfluidic device attachment point mounted to the printer extruder. A microfluidic device was mounted to the system, with the water bath being filled with DI water. The STrIPS fiber extrusion process was conducted as mentioned above, with the ternary and 100 mM NaCl solution being pumped through the inner and outer capillaries, respectively. The fiber formation was allowed to stabilize, and then the controlled deposition was initiated via the use of custom‐designed G‐Code controlling the X/Y gantry. Once scaffold fabrication was achieved, the Allevi 2 405 nM light module was used to polymerize the structure. The polymerized scaffold was removed from the water bath and subjected to 3 consecutive days of excess CTAB removal in 100% v/v ethanol, with the ethanol being replaced every day. Further, 2 days of scaffold washing were conducted in an agitated DI water bath. Scaffolds were then cut into 2 mm × 2 mm × 2 mm pieces and moved into the biosafety cabinet, sterilized with 70% v/v ethanol under UV, and left in 1× PBS until cell culture. All cell culture experiments and analyses were conducted similarly to the cell studies with single fibers. Imaging of cell‐laden scaffolds was done in both intact and ruptured conditions to assess cellular penetration, integration, and ECM deposition throughout the volume of the scaffolds.

##### ATR–FTIR Analysis of PEGDA‐BIPORES

ATR–FTIR spectroscopy in the absorbance mode from 400 to 4000 cm^−1^ (Nicolet iS10 spectrometer, Thermo Fisher Scientific) was used for the chemical analysis of the PEGDA ternary mixture and PEGDA‐BIPORES fibers.

##### Confocal Laser Scanning Microscopic Characterization of PEGDA‐BIPORES

To examine the structural features of the PEGDA‐BIPORES fibers, Nile Red was added to the fiber precursor mixture, and the collected fibers were transferred to a microscope slide. Highly detailed confocal microscopic images were generated to efficiently characterize the bicontinuous morphologies. 3D rendering and visualization of the fibers were done from confocal z‐stack scanning using the Imaris microscopic image analysis software (version 10.1, Oxford Instruments).

##### Scanning Electron Microscopic Analysis of PEGDA‐BIPORES

Surface morphology characterization of the fabricated PEGDA‐BIPORES fibers was performed using Hitachi TM‐1000 and TESCAN Vega3 SBH SEMs. SEM images were acquired to examine the surface details and features of the PEGDA‐BIPORES fibers. Fiber diameter and pore size were measured using the ImageJ software. For SEM imaging of biological samples, the cell‐laden samples were dehydrated with a graded ethanol series (50% v/v ethanol for 5 min, 70% v/v ethanol for 5 min, 80% v/v ethanol for 10 min, 95% v/v ethanol for 10 min, 100% v/v ethanol for 10 min (2 times)), followed by a hexamethyldisilane (HMDS) series (3:1 ethanol:HMDS for 15 min, 1:1 ethanol:HMDS for 15 min, 1:3 ethanol:HMDS for 15 min, 100% HMDS for 15 min (3 times)). The HMDS was allowed to evaporate overnight. The dried samples were mounted on a double‐sided carbon‐taped SEM stub and sputter coated with gold at 30 mA for 20 s prior to imaging.

##### Surface Profiling of Biomaterials

The 3D surface topography, roughness, and specific surface area of PEGDA‐BIPORES fibers (top, longitudinal, and transverse cross‐sectional surfaces), PEGDA ternary mixture, and PEGDA hydrogel were analyzed using an advanced 3D laser scanning confocal microscope capable of noncontact profile measurements (Keyence, VK‐X150). 3D rendering, visualization, and all associated measurements were done using the VK Viewer software from Keyence.

##### Contact Angle Measurements

Sessile droplet contact angle analyses were conducted for the PEGDA‐BIPORES fibers and PEGDA hydrogels using an optical goniometer (Wet Scientific) operated at room temperature. A thin sheet of PEGDA (20 wt%) containing LAP (0.25 wt%) dissolved in 1× PBS was spread on a glass slide and photocrosslinked with visible light for 60 s. 10 μL droplets of DI water were deposited on both the BIPORES fiber mesh and PEGDA hydrogels, and high‐speed digital photographs were captured. The digital photographs were analyzed using ImageJ to measure the contact angles.

##### Degradation Studies of the PEGDA‐BIPORES Fibers

The degradation tests of the PEGDA‐BIPORES fibers were carried out to generate their degradation profile, similar to previously published protocols.^[^ ^60^, ^61^ ^]^ After lyophilizing and weighing, the samples were incubated in 1 mL of 1× PBS in 1.5 mL centrifuge tubes inside a 37 °C incubator for a period of three months. The 1× PBS was changed twice weekly to maintain constant activity. At predetermined time points (1, 2, and 3 months), the 1× PBS was removed, and the fibers were lyophilized for 24 h, followed by dry weight measurements. The extent of degradation (D%) of the fibers was calculated based on the weight loss using the following equation.

(1) $$D\%=\frac{Initial\;Dry\;Weight-Dry\;Weight\;at\;time\;t}{Initial\;Dry\;Weight}\times 100$$

##### Real‐Time Assessment of Single Cell‐Biomaterial Affinity

PEGDA‐BIPORES fibers were attached to a 35 mm poly‐D‐lysine‐coated glass bottom dish (MatTek, P35GC‐1.5‐14C) using medical silicone adhesive (A‐100, Factor II, Inc.). PEGDA ternary mixture (prepared as described above) and PEGDA hydrogel solution (40% w/v PEGDA, 0.25% w/v LAP) were deposited on the glass bottom dishes and crosslinked with a 405 nm light source for 60 s. Routinely cultured hMSCs (Cell Applications Inc.) or hiPSC‐CFs were dispersed into prepared dishes filled with culture medium (Dulbecco's modified Eagle medium (DMEM)/F‐12 (Gibco), 15% v/v fetal bovine serum (FBS) (Gibco), and 1% v/v penicillin‐streptomycin (P/S) or supplemented fibroblast growth media (FGM)‐3 (PromoCell)).

A custom‐made laser tweezer system (Figure S5, Supporting Information) was used to form an optical trap at the stage of an inverted microscope (Nikon, Ti‐Eclipse). A beam expander was used to expand a beam of light from a 1064 nm laser (Coherent, Prisma 1064‐8‐V). A combination of a half‐wave plate and a polarizing beam splitter was used to reduce the intensity of the expanded beam before entering the microscope objective. A dichroic mirror (Chroma, Z900DCSP) reflected and directed the laser beam through the back aperture of a 100× oil immersion objective (Nikon Plan Fluor, NA = 1.3). The objective focused the light onto the microscope stage, creating the optical trap. A 100 W halogen lamp source was used to illuminate the sample. The light from the sample was reflected using a mirror and focused onto an electron multiplying‐charge‐coupled device (EM‐CCD) camera (Hamamatsu C9100) to form the image of the sample. The images and videos were recorded using HCImage software.

Optically trapped live hMSCs or hiPSC‐CFs were tested for their affinity toward the three biomaterials in two different configurations, tapping mode and contact mode. In tapping mode, an optically trapped hMSC or hiPSC‐CF was placed 10 μm away from the biomaterial. The biomaterial, attached to the bottom of the dish, was brought in contact with the trapped cell five times using a piezoelectric translation stage (PZT) (Physik Instrumente, model *P*‐527.C3). The time of interaction for each contact was 1 s. In contact mode, the biomaterial was brought in contact with an optically trapped hMSC or hiPSC‐CF, 10 μm away from the biomaterial, two times using PZT. The time of interaction for the first contact was 5 s, and for the second contact was 10 s. In both configurations, the biomaterial was displaced toward and away from a trapped hMSC or hiPSC‐CF at a constant velocity of 10 mm s^−1^. All experiments were performed within 2 h of cell dispersion into prepared dishes.

##### Estimation of Cell–Biomaterial Adhesion Force

An optically trapped cell experiences a trapping force perpendicular to the axis of propagation of the laser beam. This transverse trapping force is governed by the equation

(2) F=−kx

where *F* is the force experienced by a trapped cell displaced by a distance *x* from the trap center, and *k* is the trap stiffness. The trap stiffness was calibrated using known viscous drag forces on an optically trapped bead and was estimated at 319.86 pN μm^−1^. Based on Equation (2), the maximum force (*F*_max_) occurs when the trapped cell is displaced by a distance equivalent to the cell radius (r). The cell escapes the trap if an external force overcoming *F*_max_ is applied to the cell. The minimum force of adhesion between a cell and a biomaterial, providing the external force, can be estimated when a cell escapes the optical trap due to its interaction with the biomaterial.

##### Cell Culture on PEGDA‐BIPORES Scaffolds

Assessment of the compatibility of the PEGDA‐BIPORES fibers with different types of cells was done in a fashion similar to protocols previously reported.^[^ ^60^, ^61^ ^]^ 50000 cells were seeded on the PEGDA‐BIPORES fibers in 48‐well plates (Fisherbrand, Fisher Scientific) with the cell‐specific growth medium. For C2C12 (Cell Applications Inc.), the medium was composed of DMEM (Gibco) with 15% v/v FBS and 1% v/v P/S. For hMSCs, DMEM/F‐12 medium with 15% v/v FBS and 1% v/v P/S was used. The cultures were maintained at 37 °C in a humidified 5% CO_2_ incubator for different time points, with fresh culture media every 48 h.

##### Osteogenic Differentiation of hMSCs on PEGDA‐BIPORES Scaffolds

The hMSCs were expanded in a growth medium composed of DMEM/F12 supplemented with 15% v/v FBS and 1% v/v P/S. Cells between passages 5 and 7 were used for osteogenic differentiation on PEGDA‐BIPORES scaffolds based on a published protocol.^[^ ^62^ ^]^ Briefly, hMSCs were inoculated on PEGDA‐BIPORES single fibers and multi‐fibrous meshwork scaffolds at 25000 and 50000 cells, respectively. The hMSCs were pre‐cultured in the growth medium containing 100 ng mL^−1^ basic fibroblast growth factor (bFGF) (Reprocell) for 2 days after seeding, followed by medium change to osteogenic differentiation medium, formulated with low‐glucose (1 g L^−1^) DMEM (Gibco) containing 10% v/v FBS, 50 μg/mL L‐ascorbic acid (Sigma‐Aldrich), 10 mM β‐glycerophosphate (Sigma‐Aldrich), 100 nM dexamethasone (Sigma‐Aldrich), and 1% v/v P/S. The differentiation medium was changed every 2 days, and characterization of osteogenic differentiation was carried out on days 14 and 21.

##### Differentiation of hiPSCs to Cardiomyocytes (hiPSC‐CMs)

WTC‐11 hiPSCs (AICS, Passage #40) were differentiated into cardiomyocytes (hiPSC‐CMs) through an established protocol.^[^ ^63^ ^]^ Briefly, hiPSCs were cultivated in a feeder‐free environment on 1% v/v Geltrex (Gibco)‐coated tissue culture plates until they reached a confluence of ≈75%–80%. Subsequently, a precisely controlled cardiomyocyte differentiation process was initiated using specialized small molecules, namely 10 μM CHIR99021 (Cayman Chemical) and 7.5 μM IWP2 (Tocris Bioscience), in Roswell Park Memorial Institute (RPMI)‐1640 media (Gibco) supplemented with insulin‐minus B27 (Gibco) (RPMI/B27(‐)), with each treatment lasting 48 h. The introduction of CHIR99021 on day 0 of the differentiation protocol inhibited the glycogen synthase kinase‐3 (GSK) pathway, triggering activation of the canonical Wnt signaling pathway. Conversely, after exactly 48 h of CHIR99021 treatment, IWP2 treatment played a pivotal role by inhibiting the Wnt pathway within the cells. Following this initiation phase, a 48‐hour recovery period in RPMI/B27(‐) medium was allowed. Growth and maturation of differentiated hiPSC‐CMs were sustained by transitioning to RPMI‐1640 medium supplemented with insulin‐containing B27 (Gibco) and 1% v/v P/S (RPMI/B27(+)) until the hiPSC‐CMs exhibited synchronized contractions. This developmental stage typically manifests around days 10–12 of differentiation. Further enrichment of the hiPSC‐CMs through a glucose starvation regimen was done utilizing a glucose‐free RPMI/B27(+) medium with 4 mM sodium lactate (Gibco) from day 13 to day 19 of the protocol, with medium changes every 3 days. On day 19, the RPMI/B27(+) medium was re‐employed to allow for cell recovery. Subsequently, hiPSC‐CMs were dissociated using TrypLE Express (Gibco) and used for further experiments.

##### Differentiation of hiPSCs to Cardiac Fibroblasts (hiPSC‐CFs)

The hiPSC‐CFs differentiation protocol is published elsewhere.^[^ ^64^ ^]^ For obtaining hiPSC‐CFs, hiPSCs were treated with RPMI/B27(‐) containing 7 μM CHIR99021 for one day (day 0), followed by a day of RPMI/B27(‐) treatment for cell recovery (day 1). On day 2, cells were subjected to the hiPSC‐CF differentiation medium, formulated with high‐glucose (4.5 g L^−1^) DMEM (Gibco), supplemented with human serum albumin (500 μg mL^−1^), linoleic acid (0.6 μM), lecithin (0.6 μg mL^−1^), and bFGF (70 ng mL^−1^). This differentiation medium was changed every 48 h until day 20. On day 20, the hiPSC‐CFs were dissociated using 0.05% v/v trypsin/EDTA and replated on uncoated plates containing FGM‐3. The FGM‐3 medium was changed every 48 h until the hiPSC‐CFs reached about 80%−85% confluency and subsequently passaged. hiPSC‐CFs between passage numbers 5 and 7 were used for further experiments.

##### Co‐culture of hiPSC‐CMs and hiPSC‐CFs on PEGDA‐BIPORES Scaffolds

The hiPSC‐CMs and hiPSC‐CFs were co‐cultured on the PEGDA‐BIPORES biomaterial at a ratio of 4:1, respectively. 100000 hiPSC‐CMs and 25000 hiPSC‐CFs were seeded on the PEGDA‐BIPORES fibers in each well of a 48‐well plate. A medium composed of RPMI/B27 + and complete FGM‐3 mixed at a 1:1 ratio was used for the co‐culture, which was changed every 2 days. The co‐culture was continued at 37 °C in a humidified 5% CO_2_ incubator for 12 days, during which synchronous beating of the hiPSC‐CMs as well as the characteristic morphology of the hiPSC‐CFs became prominent.

##### Cytocompatibility Assays of PEGDA‐BIPORES Scaffolds

To assess the health of the cells cultured on the PEGDA‐BIPORES fibers or meshwork, a calcein‐AM/ethidium homodimer‐I‐based live/dead cell viability kit from Invitrogen was used following the product manual. The cells were stained with a solution of 2 μM of calcein‐AM and 4 μM of ethidium homodimer‐I in 1× Dulbecco's PBS for 30 min at room temperature on days 1, 4, 7, and 14, and fluorescent microscopic images were captured using a Zeiss LSM 880 inverted confocal microscope. Live cells were labeled green, while dead cells were labeled red. The ImageJ software was utilized to quantify the percentage of live cells out of the total number of cells, for determining cell viability.

The cellular metabolism was assessed on days 1, 4, 7, and 14 following cell seeding using the PrestoBlue cell viability reagent (Invitrogen) according to the manufacturer's protocol. The cells were incubated in culture media with 10% v/v PrestoBlue reagent for 3 h at 37 °C, followed by fluorescence measurement (excitation at 560 nm, emission at 610 nm) using a Tecan fluorescence plate reader. PrestoBlue‐containing culture media were used to establish background fluorescence.

To visualize cellular cytoskeletal spreading and nuclear proliferation on the PEGDA‐BIPORES, fluorescent phalloidin (conjugated with AlexaFluor 488) labeling of cytoskeletal actin filaments and DAPI (Biotium) staining of cell nuclei were performed. After fixing the monolayer cultures at days 1, 4, 7, and 14 in 4% v/v paraformaldehyde (Thermo Fisher Scientific) for 15 min, the samples were permeabilized in 0.1% Triton X‐100 (Sigma‐Aldrich) for 20 min and blocked in 1% w/v bovine serum albumin (BSA, Merck) for 45 min. Three 1× PBS washes, for 5 min each, were done after the fixation and permeabilization steps. Alexa‐fluor 488‐labeled phalloidin (1/100 dilution in 0.1% w/v BSA) was then applied to the samples for 45 min. Following three successive washes with 1× PBS, the samples were counterstained with 1 μL mL^−1^ DAPI in 1× PBS for 5 min. The samples were washed with 1× PBS once and stored in 1× PBS until microscopic imaging. Fluorescent images were acquired using a Zeiss LSM 880 inverted confocal microscope, and the ImageJ software was utilized to quantify cellular proliferation by counting the DAPI‐stained nuclei.

##### Alizarin Red S and Alkaline Phosphatase Staining for Assessment of Osteogenic Differentiation

ARS (Sigma‐Aldrich, A5533) and ALP (Sigma‐Aldrich, SCR004) staining of differentiated hMSCs growing on the PEGDA‐BIPORES fibers were done to assess the osteoblast mineralization (calcium deposits) and ALP activity, respectively. For ARS staining, the cells were fixed in 4% v/v paraformaldehyde for 30 min at room temperature. Following washing with 1× PBS twice, the samples were incubated in 40 mM ARS staining solution (pH adjusted to 4.2) for 30 min at room temperature in the dark. The samples were washed once with 1× PBS and stored in 1× PBS until imaging. For ALP staining, the staining solution was prepared by mixing fast red violet, naphthol AS‐BI phosphate solution, and water in a 2:1:1 ratio. The cells were fixed in 4% v/v paraformaldehyde for 1–2 min at room temperature. After washing the samples two times with 1× PBS, the cells were incubated in the freshly prepared ALP staining solution for 15–30 min at room temperature in the dark. The samples were washed once with 1× PBS and stored in 1× PBS until imaging. Imaging of the ARS and ALP‐stained samples was done using a Nikon Eclipse Ti phase‐inverted microscope in color mode.

##### Immunostaining for Osteogenic Biomarkers

Immunofluorescent staining of differentiated hMSCs growing on the PEGDA‐BIPORES fibers or meshwork was done to assess the presence of osteogenic differentiation biomarkers, specifically OPN and OCN. After fixing the hMSCs with 4% v/v paraformaldehyde for 15 min at room temperature on days 14 and 21 of differentiation, the samples were washed with 1× PBS, permeabilized with 0.3% v/v Triton X‐100 for 15 min, and blocked with 1% v/v BSA in 1× PBS containing 0.1% v/v Triton X‐100 for 1 h. Subsequently, the samples were stained overnight with primary antibodies against OPN (Proteintech, 22 952‐1‐AP, 1:200 dilution in blocking buffer) and OCN (Proteintech, 23 418‐1‐AP, 1:200 dilution in blocking buffer) at 4 °C. After washing three times with 1× PBS, the samples were incubated with an Alexa Fluor 488‐conjugated goat anti‐rabbit IgG (H + L) highly cross‐adsorbed secondary antibody (Invitrogen, A‐11 034, 1:500 dilution) for 1 h at room temperature. Following three washes with 1× PBS, the samples were counterstained with 1 μL mL^−1^ DAPI in 1× PBS for 5 min at room temperature. The fluorescent imaging was carried out using a Zeiss LSM 880 inverted confocal microscope.

##### Immunostaining for Cardiac Biomarkers

Immunofluorescent staining of differentiated hiPSC‐CMs or hiPSC‐CFs cultured on the PEGDA‐BIPORES biomaterial was done to assess the expression of CM and CF biomarkers, specifically SAA, cTnT, vimentin, and TE‐7. After fixing the hiPSC‐CMs or hiPSC‐CFs cultured on PEGDA‐BIPORES biomaterials with 4% v/v paraformaldehyde for 15 min at room temperature, the samples were washed three times with ice‐cold 1× PBS. Permeabilization with 0.3% v/v Triton X‐100 was done for 15 min, followed by three washes with 1× PBS for 5 min each. The samples were blocked with 1% v/v BSA in 1× PBS containing 0.1% v/v Triton X‐100 for 1 h. Subsequently, the samples were incubated overnight with primary antibodies against SAA (Sigma‐Aldrich, A7811, 1:400 dilution), cTnT (Abcam, ab45932, 1:400 dilution), vimentin (Cell Signaling Technology, #5741, 1:200 dilution) or TE‐7 (Sigma‐Aldrich, CBL271, 1:200 dilution) in the blocking buffer at 4 °C. After washing three times with 1× PBS for 5 min each, the SAA or the TE‐7 samples were incubated with an Alexa Fluor 594‐conjugated goat anti‐mouse IgG (H + L) highly cross‐adsorbed secondary antibody (Invitrogen, A‐11 032, 1:500 dilution), and the cTnT or the vimentin samples were incubated with an Alexa Fluor 488‐conjugated goat anti‐rabbit IgG (H + L) highly cross‐adsorbed secondary antibody (Invitrogen, A‐11 034, 1:500 dilution), diluted in the blocking buffer, for 1 h in the dark at room temperature. Following three washes with 1× PBS for 5 min each, the samples were counterstained with 1 μL mL^−1^ DAPI in 1× PBS for 5 min at room temperature. The fluorescent imaging was carried out using a Zeiss LSM 880 inverted confocal microscope.

##### Co‐immunostaining for Cardiac Biomarkers

Immunofluorescent co‐staining of co‐cultured hiPSC‐CMs and hiPSC‐CFs on the PEGDA‐BIPORES scaffolds was done to assess the expression of cardiac biomarkers. SAA and vimentin antibodies were paired together, while cTnT and TE‐7 antibodies were used together to stain the co‐cultured samples. The fixing, permeabilization, blocking, and washing protocols, as well as the primary and secondary antibodies and their dilutions, were the same as mentioned for cardiac biomarkers immunostaining above. For the primary antibody incubations, one set of samples was sequentially incubated overnight at 4 °C with the antibodies against SAA and then with the antibodies against vimentin, while for the other set of samples, the antibodies against cTnT were used, followed by the antibodies against TE‐7. The samples were washed between the two primary antibody incubations three times with 1× PBS for 5 min each. For the secondary antibody incubation, the SAA/vimentin samples were sequentially incubated in Alexa Fluor 594‐conjugated anti‐mouse (for SAA), followed by Alexa Fluor 488‐conjugated anti‐rabbit (for vimentin) secondary antibodies for 1 h each. The cTnT/TE‐7 samples were first incubated in Alexa Fluor 488‐conjugated anti‐rabbit (for cTnT), followed by Alexa Fluor 594‐conjugated anti‐mouse (for TE‐7) secondary antibodies for 1 h each. The samples were washed between the two secondary antibody incubations three times with 1× PBS for 5 min each. Finally, all the samples were counterstained with 1 μL mL^−1^ DAPI in 1× PBS for 5 min at room temperature. The fluorescent microscopic imaging was carried out using a Zeiss LSM 880 inverted confocal microscope.

##### Subcutaneous Implantation of PEGDA‐BIPORES Scaffolds

The animal experiments conducted for this study were reviewed and approved by the IACUC (protocol #75) at the University of California, Riverside, conforming to appropriate protocols and rules. 10 to 12‐week‐old male Wistar rats weighing 200–225 grams were sourced from Charles River (Boston, MA, USA) and kept in separate cages in the vivarium under standard circadian day/night cycles with food and water as and when required. The animals were anesthetized with 4%‐5% isoflurane for 1–2 min at a flow rate of 2–3.5 L min^−1^ in an induction chamber, and the adequacy of anesthesia was determined by the absence of a pedal reflex upon toe pinching every 5 min. The anesthesia was then maintained at 2% isoflurane delivered through a nose cone. During anesthesia and surgery, heat support was provided continuously by a heating pad underneath the sterile surgical pad to prevent body temperature drop and protect against potential mortality. Proparacaine hydrochloride ophthalmic solution (0.5%, Sandoz Inc., Princeton, NJ) was applied to the rat's eyes to prevent damage after anesthesia. Hair on the dorsal skin was removed by shaving under isoflurane, and an analgesic, Meloxicam‐SR (sustained release, 2.5 mg Kg^−1^), was administered subcutaneously 30 min before the surgery. The surgical site was thoroughly disinfected using 70% v/v isopropyl alcohol (3 times), followed by 2% v/v chlorhexidine scrub (3 times) and finally once with povidone‐iodine to provide adequate and long‐lasting disinfection. In a step‐wise process, six 1 cm incisions were made on the posterior mediodorsal skin to form lateral subcutaneous pockets using surgical scalpels. PEGDA‐BIPORES fibers measuring around 5 mm in length were then carefully implanted into four of these pockets. The remaining two pockets did not receive any biomaterial and served as controls.  Subsequently, the surgical incisions were sutured, and the animals were allowed to recover from anesthesia. Animals were euthanized using exsanguination and cervical dislocation on days 3, 7, 14, and 28 after implantation. Skin tissues with or without the implanted fibers were extracted and placed in 1× PBS for further analysis.

##### Histological analysis and immunofluorescent staining

To examine the host immune response elicited by the implanted PEGDA‐BIPORES fibers, histological analyses were carried out on cryosections of the excised tissue samples, similar to previously reported methods.^[^ ^60^, ^61^ ^]^ The samples were fixed in 4% v/v paraformaldehyde for 4 h, followed by serial incubation in 15% w/v and 30% w/v sucrose at 4 °C until the tissues sank to the bottom. The samples were flash‐frozen in liquid nitrogen following optimal cutting temperature (OCT) embedding. The frozen OCT‐embedded samples were sectioned using a Leica CM1950 Cryostat, producing 7–15 μm thick cryosections, which were mounted on poly‐L‐lysine‐coated slides. These slides underwent H&E (Abcam, ab245880) histological staining as per the product guidelines to characterize the inflammatory response. The H&E‐stained samples were imaged using a Nikon Eclipse Ti phase inverted microscope in color mode. The cryosections were also immunofluorescently labeled using the primary antibody anti‐CD68 (ab283654, 1:100 dilution) from Abcam and Alexa Fluor 594‐conjugated secondary antibody (ab150080, 1:200 dilution) for detection. All samples were counterstained with DAPI, and fluorescence microscopic imaging was done using a Zeiss LSM 880 inverted confocal microscope.

##### Statistical analysis

A minimum of 3 biological replicates were used for all the experiments. The data were subjected to a Student's *t*‐test for two‐group comparisons or one‐way ANOVA with Tukey's post‐hoc test for multiple‐group comparisons using IBM SPSS Statistics software (version 28). All data are represented as the mean ± standard deviation (SD) or standard error of the mean (SEM) of measurements. Statistical significance levels were as follows: ns–non‐significant, *p < 0.05, **p < 0.01, ***p < 0.001.

## Conflict of Interest

The authors declare no conflict of interest.

## Supporting information

Supplementary Material

## Data Availability

The data that support the findings of this study are available from the corresponding author upon reasonable request.

## References

[1] T. L. Place , F. E. Domann , A. J. Case , Free Radical Biol. Med. 2017, 113, 311. 29032224 10.1016/j.freeradbiomed.2017.10.003 PMC5699948

[2] D. S. Masson‐Meyers , L. Tayebi , J. Tissue Eng. Regen. Med. 2021, 15, 747. 34058083 10.1002/term.3225 PMC8419139

[3] M. Soltani , M. A. Maleki , A. H. Kaboodrangi , B. Mosadegh , Chem. Eng. Sci. 2018, 184, 1.

[4] F. J. Maksoud , M. F. Velázquez De La Paz , A. J. Hann , J. Thanarak , G. C. Reilly , F. Claeyssens , N. H. Green , Y. S. Zhang , J. Mater. Chem. B 2022, 10, 8111. 36205119 10.1039/d1tb02628c

[5] Q. L. Loh , C. Choong , Tissue Eng., Part B 2013, 19, 485. 10.1089/ten.teb.2012.0437 PMC3826579 23672709

[6] T. Liu , Y. Wang , T. Kuang , Macro Mater. Eng. 2024, 309, 2300246.

[7] H. Chen , Q. Han , C. Wang , Y. Liu , B. Chen , J. Wang , Front. Bioeng. Biotechnol. 2020, 8, 609. 32626698 10.3389/fbioe.2020.00609 PMC7311579

[8] L. Zhu , D. Luo , Y. Liu , Int. J. Oral Sci. 2020, 12, 6. 32024822 10.1038/s41368-020-0073-y PMC7002518

[9] S. Liu , J.‐M. Yu , Y.‐C. Gan , X.‐Z. Qiu , Z.‐C. Gao , H. Wang , S.‐X. Chen , Y. Xiong , G.‐H. Liu , S.‐E. Lin , A. McCarthy , J. V. John , D.‐X. Wei , H.‐H. Hou , Mil. Med. Res. 2023, 10, 16. 36978167 10.1186/s40779-023-00448-w PMC10047482

[10] E. M. Sussman , M. C. Halpin , J. Muster , R. T. Moon , B. D. Ratner , Ann. Biomed. Eng. 2014, 42, 1508. 24248559 10.1007/s10439-013-0933-0

[11] R. Sridharan , A. R. Cameron , D. J. Kelly , C. J. Kearney , F. J. O’Brien , Mater. Today 2015, 18, 313.

[12] P. Xia , Y. Luo , J. Biomed. Mater. Res. 2022, 110, 1206. 10.1002/jbm.b.34979 34860454

[13] H. Mehdizadeh , S. Sumo , E. S. Bayrak , E. M. Brey , A. Cinar , Biomater. 2013, 34, 2875. 10.1016/j.biomaterials.2012.12.047 23357368

[14] D. Y. Kwon , J. Y. Park , B. Y. Lee , M. S. Kim , Polymers 2020, 12, 2210.32993178

[15] Y.‐C. Chiu , J. C. Larson , A. Isom , E. M. Brey , Tissue Eng., Part C 2010, 16, 905. 10.1089/ten.TEC.2009.0646 19905877

[16] Y. Chen , Y. Li , X. Wang , X. Mo , Y. Chen , Z. Deng , X. Ye , J. Yu , ACS Appl. Mater. Interfaces 2024, 16, 61664. 39474891 10.1021/acsami.4c13119

[17] J. Ding , J. Zhang , J. Li , D. Li , C. Xiao , H. Xiao , H. Yang , X. Zhuang , X. Chen , Prog. Polym. Sci. 2019, 90, 1.

[18] K. Prem Ananth , N. D. Jayram , Annals of 3D Printed Med. 2024, 13, 100141.

[19] P. A. Lovell , F. J. Schork , Biomacromolecules 2020, 21, 4396. 32543173 10.1021/acs.biomac.0c00769

[20] A. Tamayol , M. Akbari , N. Annabi , A. Paul , A. Khademhosseini , D. Juncker , Biotechnol. Adv. 2013, 31, 669. 23195284 10.1016/j.biotechadv.2012.11.007 PMC3631569

[21] M. S. B. Reddy , D. Ponnamma , R. Choudhary , K. K. Sadasivuni , Polymers 2021, 13, 1105. 33808492 10.3390/polym13071105 PMC8037451

[22] D. F. Williams , Front. Bioeng. Biotechnol. 2019, 7, 127. 31214584 10.3389/fbioe.2019.00127 PMC6554598

[23] M. N. Lee , J. H. J. Thijssen , J. A. Witt , P. S. Clegg , A. Mohraz , Adv. Funct. Mater. 2013, 23, 417.

[24] M. Reeves , K. Stratford , J. H. J. Thijssen , Soft. Matter. 2016, 12, 4082. 27035101 10.1039/c5sm03102h

[25] S. Gam , A. Corlu , H.‐J. Chung , K. Ohno , M. J. A. Hore , R. J. Composto , Soft. Matter. 2011, 7, 7262.

[26] M. F. Haase , K. J. Stebe , D. Lee , Adv. Mater. 2015 44/2015. 27, 7013. 10.1002/adma.201503509 26437299

[27] S. P. Kharal , R. P. Hesketh , M. F. Haase , High‐Tensile Strength , Adv. Funct. Mater. 2020, 30, 2003555.

[28] G. Di Vitantonio , T. Wang , M. F. Haase , K. J. Stebe , D. Lee , ACS Nano 2019, 13, 26. 30525442 10.1021/acsnano.8b05718

[29] S. Cha , H. G. Lim , M. F. Haase , K. J. Stebe , G. Y. Jung , D. Lee , Sci. Rep. 2019, 9, 6363. 31019261 10.1038/s41598-019-42769-8 PMC6482178

[30] M. A. Khan , A. J. Sprockel , K. A. Macmillan , M. T. Alting , S. P. Kharal , S. Boakye‐Ansah , M. F. Haase , Adv. Mater 2022, 34, 2109547. 10.1002/adma.202109547 35305279

[31] X. Wang , T. Lou , W. Zhao , G. Song , C. Li , G. Cui , J. Biomater. Appl. 2016, 30, 1545. 26945811 10.1177/0885328216636320

[32] V. D. Ranjan , P. Zeng , B. Li , Y. Zhang , Biomater. Sci. 2020, 8, 2175. 32091515 10.1039/c9bm01986c

[33] C.‐D. Kim , K.‐M. Koo , H.‐J. Kim , T.‐H. Kim , Biosens. Basel 2024, 14, 407.

[34] A. A. Dayem , H. Y. Choi , G. Yang , K. Kim , S. K. Saha , J. Kim , S. Cho , Biotechnol. J. 2016, 11, 1550. 27797150 10.1002/biot.201600453

[35] L. Andrée , D. Barata , P. Sutthavas , P. Habibovic , S. Van Rijt , Acta Biomater. 2019, 96, 557. 31284095 10.1016/j.actbio.2019.07.008

[36] M. E. Cates , P. S. Clegg , Soft. Matter. 2008, 4, 2132.

[37] M. F. Haase , N. Sharifi‐Mood , D. Lee , K. J. Stebe , ACS Nano 2016, 10, 6338. 27227507 10.1021/acsnano.6b02660

[38] D. R. Griffin , W. M. Weaver , P. O. Scumpia , D. Di Carlo , T. Segura , Nat. Mater. 2015, 14, 737. 26030305 10.1038/nmat4294 PMC4615579

[39] S. I. Somo , B. Akar , E. S. Bayrak , J. C. Larson , A. A. Appel , H. Mehdizadeh , A. Cinar , E. M. Brey , Tissue Eng. Part C 2015, 21, 773. 10.1089/ten.tec.2014.0454 PMC5915224 25603533

[40] J. Gu , Q. Zhang , M. Geng , W. Wang , J. Yang , A. U. R. Khan , H. Du , Z. Sha , X. Zhou , C. He , Bioact. Mater. 2021, 6, 3254. 33778203 10.1016/j.bioactmat.2021.02.033 PMC7970223

[41] W. Li , Y. Zhao , Z. Cheng , F. Niu , J. Ding , Y. Bai , Z. Li , A. C. Midgley , M. Zhu , Mater. Design 2025, 251, 113711.

[42] J. Li , W. Zhi , T. Xu , F. Shi , K. Duan , J. Wang , Y. Mu , J. Weng , Regener. Biomater. 2016, 3, 285. 10.1093/rb/rbw031 PMC5043155 27699059

[43] S. Krainer , U. Hirn , Colloids Surf., A 2021, 619, 126503.

[44] G. Strnad , N. Chirila , C. Petrovan , O. Russu , Procedia Technol. 2016, 22, 946.

[45] K. L. Menzies , L. Jones , Optom. Vision Sci. 2010, 87, 387. 10.1097/OPX.0b013e3181da863e 20375749

[46] D. Eberli , Ed., Regenerative Medicine And Tissue Engineering: Cells And Biomaterials (IntechOpen, Erscheinungsort nicht ermittelbar 2011). 10.5772/837.

[47] R. Tudureanu , I. M. Handrea‐Dragan , S. Boca , I. Botiz , IJMS 2022, 23, 7731. 35887079 10.3390/ijms23147731 PMC9315624

[48] H. S. Kim , S. G. Kumbar , S. P. Nukavarapu , Curr. Opin. Biomed. Eng. 2021, 17, 100260. 33521410 10.1016/j.cobme.2020.100260 PMC7839921

[49] L. Xiao , Y. Sun , L. Liao , X. Su , J. Mater. Chem. B 2023, 11, 2550. 36852826 10.1039/d2tb01875f

[50] H. Zheng , Y. Tian , Q. Gao , Y. Yu , X. Xia , Z. Feng , F. Dong , X. Wu , L. Sui , Front. Bioeng. Biotechnol. 2020, 8, 463. 32509748 10.3389/fbioe.2020.00463 PMC7248375

[51] L. Yin , H. Bien , E. Entcheva , Am. J. Physiol.‐Heart Circ. Physiol. 2004, 287, H1276. 15105172 10.1152/ajpheart.01120.2003

[52] V. A. Schulte , M. Díez , M. Möller , M. C. Lensen , Biomacromolecules 2009, 10, 2795. 19785405 10.1021/bm900631s

[53] C.‐Y. Yang , W.‐Y. Huang , L.‐H. Chen , N.‐W. Liang , H.‐C. Wang , J. Lu , X. Wang , T.‐W. Wang , J. Mater. Chem. B 2021, 9, 567. 33289776 10.1039/d0tb01605e

[54] X. Yang , Y. Li , X. Liu , Q. Huang , W. He , R. Zhang , Q. Feng , D. Benayahu , Biomed. Mater. 2016, 12, 015001. 27910816 10.1088/1748-605X/12/1/015001

[55] X. Yang , Y. Li , X. Liu , Q. Huang , R. Zhang , Q. Feng , Regener. Biomater. 2018, 5, 229. 10.1093/rb/rby014 PMC6077779 30094062

[56] M. T. Tavares , M. B. Oliveira , J. F. Mano , J. P. S. Farinha , C. Baleizão , Mater. Sci. Eng.: C 2020, 107, 110348. 10.1016/j.msec.2019.110348 31761176

[57] P. Beauchamp , C. B. Jackson , L. C. Ozhathil , I. Agarkova , C. L. Galindo , D. B. Sawyer , T. M. Suter , C. Zuppinger , Front. Mol. Biosci. 2020, 7, 14. 32118040 10.3389/fmolb.2020.00014 PMC7033479

[58] J. Veldhuizen , J. Cutts , D. A. Brafman , R. Q. Migrino , M. Nikkhah , Biomaterials 2020, 256, 120195. 32623207 10.1016/j.biomaterials.2020.120195

[59] T. J. Thorson , R. E. Gurlin , E. L. Botvinick , A. Mohraz , Acta Biomaterialia 2019, 94, 173. 31233892 10.1016/j.actbio.2019.06.031 PMC7433011

[60] I. Noshadi , B. W. Walker , R. Portillo‐Lara , E. Shirzaei Sani , N. Gomes , M. R. Aziziyan , N. Annabi , Sci. Rep. 2017, 7, 4345. 28659629 10.1038/s41598-017-04280-w PMC5489531

[61] V. Krishnadoss , B. Kanjilal , A. Masoumi , A. Banerjee , I. Dehzangi , A. Pezhouman , R. Ardehali , M. Martins‐Green , J. Leijten , I. Noshadi , Mater. Today Adv. 2023, 17, 100352.

[62] Y. Tai , S. Yang , S. Yu , A. Banerjee , N. V. Myung , J. Nam , Nano Energy 2021, 89, 106444.

[63] X. Lian , J. Zhang , S. M. Azarin , K. Zhu , L. B. Hazeltine , X. Bao , C. Hsiao , T. J. Kamp , S. P. Palecek , Nat. Protoc. 2013, 8, 162. 23257984 10.1038/nprot.2012.150 PMC3612968

[64] A. Patino‐Guerrero , R. D. Ponce Wong , V. D. Kodibagkar , W. Zhu , R. Q. Migrino , O. Graudejus , M. Nikkhah , ACS Biomater. Sci. Eng. 2023, 9, 944. 36583992 10.1021/acsbiomaterials.2c01290

