# Supplementary material for: Bicontinuous Interconnected Porous Biomaterials for Tissue Engineering and Regeneration
Source: Small Sci. 2025 Aug 28;5(11):2500207. doi: 10.1002/smsc.202500207 (PMC12622506; doi:10.1002/smsc.202500207)
Supplement: Supplementary file 1 — Supplementary Material [file SMSC-5-2500207-s001.zip › smsc70080-sup-0001-SuppData-S1.pdf]

Supporting Information

Bicontinuous Interconnected Porous Biomaterials for Tissue Engineering and Regeneration

Aihik Banerjee,[1,Ψ] Anjana Khanal,[2,Ψ] Prince D Okoro,[1] Shankar P Kharal,[2] Kevin Dalsania,[1] Baishali Kanjilal,[1] Shiril B Iragavarapu,[1] Yiqing Chen,[3] Janitha M Unagolla,[1] Huinan H Liu,[1] Joshua T Morgan,[1] Robert P Hesketh,[2] Arash Pezhouman,[4] Reza Ardehali,[4] Bahman Anvari,[1] Martin F Haase,[5] Iman Noshadi[1,*]

[1]Department of Bioengineering, University of California, Riverside, Riverside, CA 92521, United States

[2]Department of Chemical Engineering, Rowan University, Glassboro, NJ 08028, United States

[3]Materials Science and Engineering Program, University of California, Riverside, Riverside, CA 92521, United States

[4]Department of Medicine, Division of Cardiology, Baylor College of Medicine, Houston, TX 77030, United States

[5]Van't Hoff Laboratory of Physical and Colloid Chemistry, Department of Chemistry, Debye Institute for Nanomaterials Science, Utrecht University, Utrecht, The Netherlands

*Corresponding author. E-mail: inoshadi@ucr.edu

[Ψ]These authors contributed equally to this work.

Ternary Phase Diagram, Binodal Curve, and Critical Composition

We determined the binodal curve in a ternary phase diagram using turbidimetry. Initially, a miscible mixture of polyethylene glycol diacrylate (PEGDA) and ethanol was prepared, and water was incrementally added to this mixture until it became cloudy. This cloudiness indicates the formation of an emulsion. The critical point was identified as the ternary liquid composition at which a transition from a PEGDA-in-water (PEGDA/W) to a water-in-PEGDA (W/PEGDA) emulsion occurs. This point signifies a change in the nature of the emulsion. To determine the critical point, Nile red, a fluorescent dye, was added to immiscible ternary mixtures at different points adjacent to the binodal line. Nile red partitions to the PEGDA phase, and the fluorescence emission was analyzed to identify the critical composition. We converted the mass of each component into volume, multiplying by their densities, and the volume fraction of each component was then plotted in the ternary phase diagram. The ternary phase diagram is shown in Figure S1. The initial volume of PEGDA, water, and ethanol to identify the binodal curve is shown in the tabular form below.

PEGDA (mL)	Water (mL)	Ethanol (mL)
0.69	0.20	0.11
0.54	0.25	0.20
0.37	0.39	0.25
0.27	0.47	0.26
0.19	0.54	0.27
0.12	0.63	0.25
0.45	0.32	0.23
0.33	0.42	0.25
0.23	0.52	0.25
0.15	0.60	0.25
0.10	0.67	0.23
0.63	0.18	0.19
0.7	0.15	0.15
0.8	0.1	0.1
0.88	0.05	0.07
0.05	0.82	0.13
1.00	0.00	0.00
0.00	0.00	1.00

Table S1. Identification of the binodal curve in the ternary phase diagram of PEGDA-BIPORES.

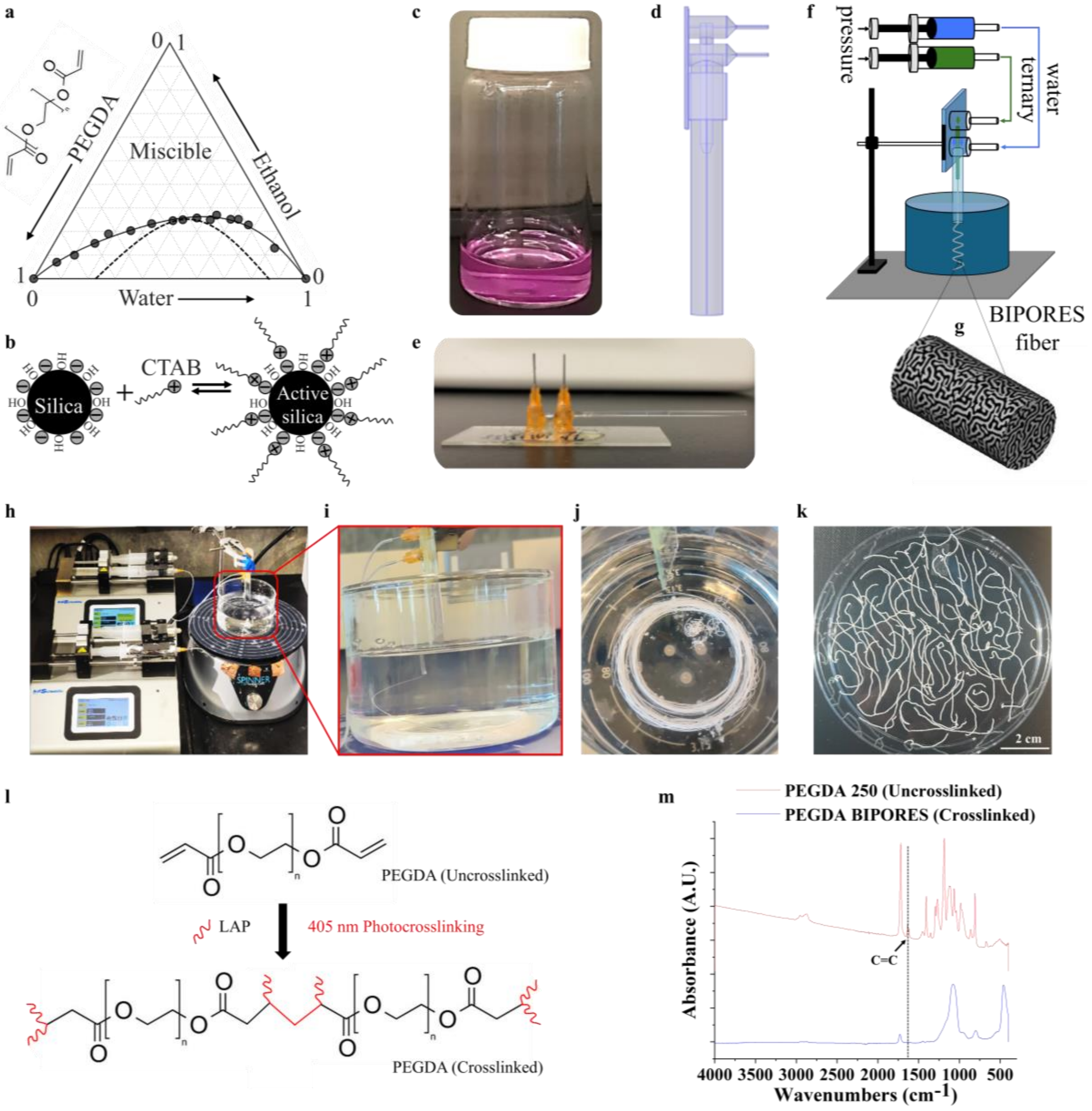

Figure S1. Formation of PEGDA-BIPORES fibers through STrIPS. (a) Equilibrium phase plot of the ternary blend of PEGDA, ethanol, and water (volume fractions). **(b)** Schematic showing functionalization of silica nanoparticles by cetyltrimethylammonium bromide (CTAB). **(c)** Photograph of the ternary liquid mixture containing CTAB, suspended nanoparticles, and lithium phenyl-2,4,6-trimethylbenzoylphosphinate (LAP) photoinitiator. Nile red dye was added for better visualization in photographs. **(d, e)** Illustrative depiction of a microfluidic BIPORES fiber extrusion device and a digital photograph of the custom-built microfluidic device made of coaxially aligned glass capillaries (inner capillary ID 600 μm with a tapered cross-section of diameter 200-300 μm, outer capillary ID 1050 μm). (**f)** Illustration of BIPORES fiber production in a microfluidic system featuring coaxial capillaries, where the fiber precursor mixture flows from the inner capillary into a water stream containing 100 mM NaCl in the outer capillary. **(g)**

Schematic of a BIPORES fiber showing the bicontinuous PEGDA-water phase morphology stabilized by interfacially jammed silica nanoparticles. **(h)** Photograph of the custom-built BIPORES fiber synthesis and collection setup consisting of two syringe pumps injecting the ternary mixture and 100 mM NaCl into the inner and outer capillaries, respectively, of the microfluidic device held in place using a finger clamp over a glass bowl filled with DI water, which is rotated using the turntable during fiber synthesis and collection. All extrusions were carried out at an injection rate of 5 mL h^{-1} for the fiber precursor mixture and 0.7 mL min^{-1} for the continuous water phase. Photographs of PEGDA-BIPORES fibers **(i, j)** during extrusion and **(k)** after photocrosslinking. (**l)** Reaction scheme for photocrosslinking of PEGDA using LAP as the photoinitiator. (**m)** ATR-FTIR spectra of uncrosslinked PEGDA (M_n 250) and crosslinked PEGDA-BIPORES fibers, showing the disappearance of the C=C peak at ~1640 cm^{-1} following LAP-mediated 405 nm photocrosslinking.

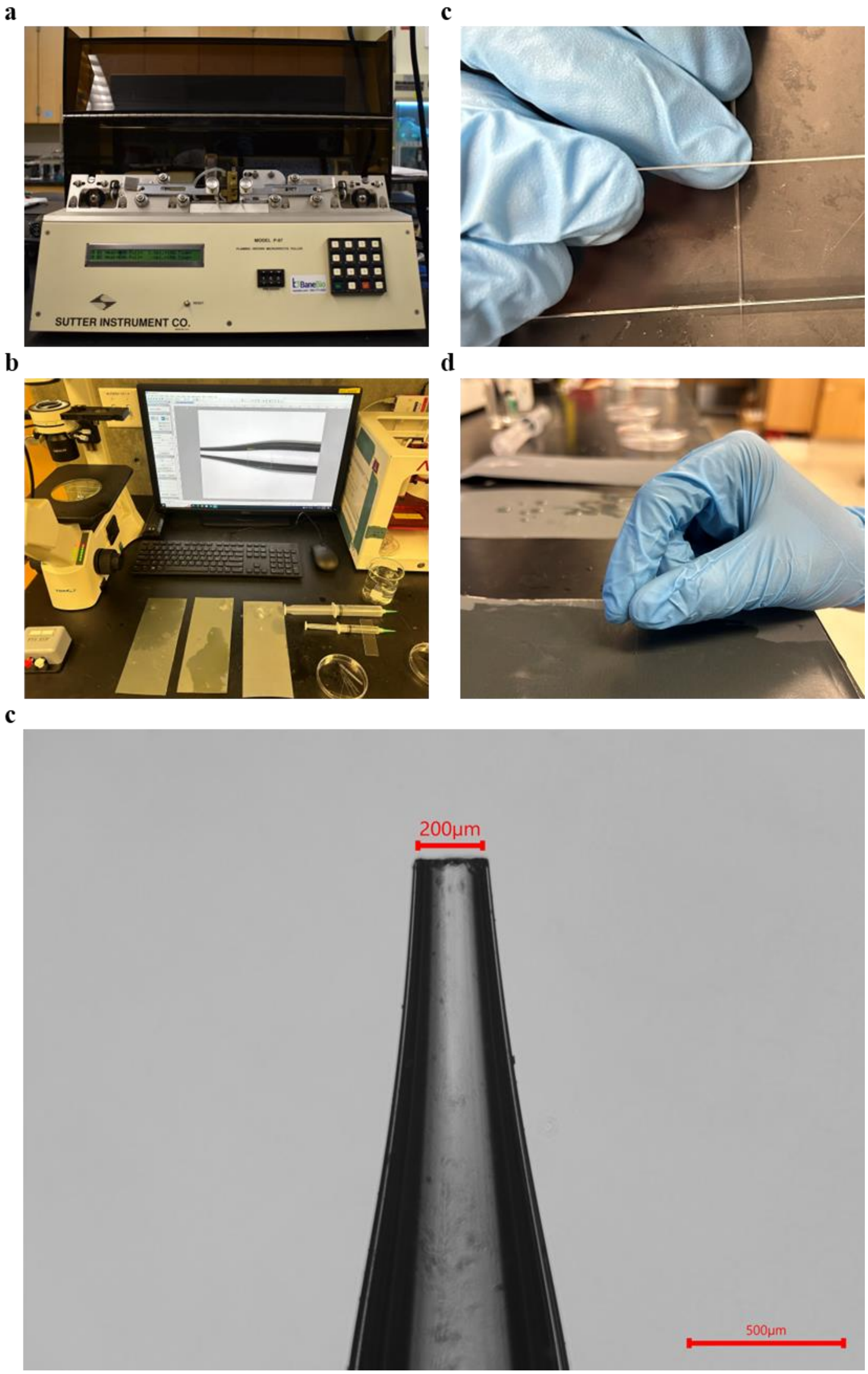

Figure S2. Inner capillary production. (a) Borosilicate glass capillaries [OD 840 μm, ID 600 μm; round] were tapered on a microcapillary puller [Sutter Instrument P27 Flaming Glass Capillary Puller] with the settings of 600 pressure and 150 pull velocity. **(b)** Capillary polishing workstation with 1500 grit, 2000 grit, and 2500 grit sandpaper. Additional equipment includes a

10 mL water purge syringe and a 50 mL air purge syringe. **(c)** Inner capillary cut via the use of a glass slide. **(d)** Capillary finishing is being done on sandpaper grits 1500, 2000, and 2500. **(e)** Brightfield microscopic image of a final tapered and finished capillary showing a tip diameter of 200 µm.

Capillary Production

Inner capillaries were initially tapered with the use of a Sutter Instrument P27 microcapillary puller with the settings 600 pressure and 150 pull velocity. The tapered capillaries were then scored and cut using a glass slide edge held at a 45-degree angle and by making a quick linear motion while applying light pressure. Alternatively, a 3D printed scoring block was used in which the capillary was scored by rotating it and then snapped by drawing the glass slide across. The cut capillaries were polished via the use of 1500 grit, 2000 grit, and 2500 grit wet and dry sandpaper. The paper was soaked before usage, with excess water removed. Sanding was done by hand, taking care to do small circular motions and keeping the capillary perpendicular to the sandpaper. Periodically throughout the sanding process, the capillary was observed under a microscope with 4X magnification to track progress. During the imaging process, great care was taken to rotate the capillary and get images from all sides of the capillary to verify its condition. Before imaging, the capillary was flushed with 10 mL of DI water and cleaned with 30 mL of air injected into the capillary with an 18-gauge syringe tip connected to Luer lock syringes. Final polishing was conducted with 2500 grit and verified to be between the diameter range of 200 µm to 300 µm. Capillaries that were considered successfully polished have minimal to no chipping on the capillary tip, fall within the diameter range of 200 µm to 300 µm, and are relatively flat with minimal angling to one side.

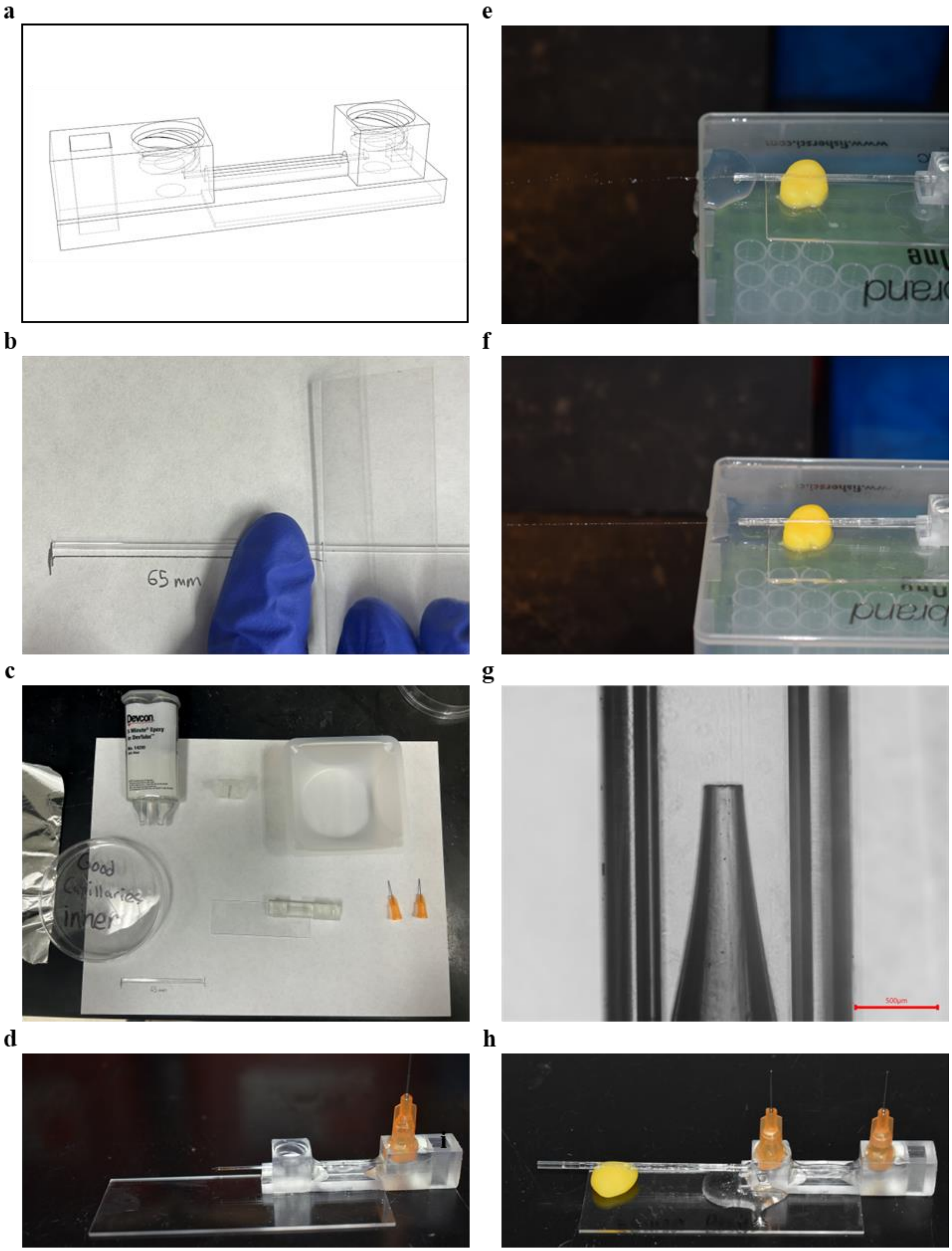

Figure S3. Microfluidic device production. (a) CAD model of microfluidic device blank designed in Autodesk Fusion 360 (2024). The device features a cutout for a microscope slide to be attached to the bottom, a Luer lock fitting for conventional syringe tips, an alignment channel for the inner capillary, and a mounting point for the synthesis clamp. **(b)** Outer capillaries [OD 1500

μm, ID 1050 μm; square] measured, scored, and cut via the use of a glass microscope slide edge. **(c)** Workstation and equipment for microfluidic device construction, including prepared inner capillaries, Devcon No. 14250 epoxy, 23-gauge syringe tips, glass slides, and microfluidic device blanks. **(d)** Microfluidic device with just the inner capillary and rear syringe tip assembled. **(e)** Flow testing before outer capillary alignment showing disturbed flow. **(f)** Flow testing after outer capillary alignment showing straight undisturbed flow. **(g)** Microfluidic device capillary centering image taken with an inverted microscope at 4X magnification. **(h)** Final completed microfluidic device.

Microfluidic Device Production

Before microfluidic device construction, custom-designed 3D printed blanks were prepared via the use of a resin printer. Multiple printers and resins were successfully used for this process, including a commercial-grade Form 3+ Formlabs Resin printer with Formlabs Clear V4 Resin and a hobbyist-grade Elegoo Mars 4 with Siriya Tech Fast Grey Resin. Post-processing of the printed structures involved a 30-minute rinse cycle in a vortexed 95% ethanol bath. Subsequently, the prints were removed and placed under a rotating curing platform for a 6-minute curing cycle while leaving the prints standing up. Great care was taken to space out the printed structure to allow for even curing of all blanks. Outer capillary production was conducted by identifying a target length and marking a surface. One side of the square capillary was scored via a sawing motion of a glass slide edge held at 45 degrees. Subsequent microfluidic device production comprised three crucial steps: assembly of the inner capillary system, adjustment of the outer capillary, and final assembly of the outer capillary. The inner capillary system assembly involved the use of Devcon No. 14250 2-part epoxy, where the device blank was glued onto a microscope slide, the inner capillary was inserted and glued, and the rear syringe tip was glued. During assembly, care was taken to ensure the inner capillary met the specifications described in Figure S2f, protruded halfway through the rear syringe tip port, and was seated thoroughly in its channel. Epoxy was generously allowed to flow around all holes. The blank was left to dry for 15 minutes, and then a flow test was conducted.

A successful flow test was visualized by a clear, undisturbed stream for the first 150 mm of extrusion. The outer capillary was then inserted into the system, and a supporting 1 cm ball of Play-Doh was placed below the capillary at the front of the microscope slide. Water was injected into the rear syringe port with adjustments being made to the outer capillary till a smooth, clean flow was observed. Once ideal flow was achieved, the outer capillary syringe tip was installed along with copious amounts of 2-part epoxy applied to glue the outer capillary. After 24 hours, once the epoxy had set, the supporting Play-Doh was removed. Finally, capillary coating was conducted by injecting 0.5% v/v PDADMAC and 0.5 M NaCl via a syringe pump at a flow rate of 1 mL/hr over 24 hours. The coated device was flushed thoroughly with water and used for fiber extrusion.

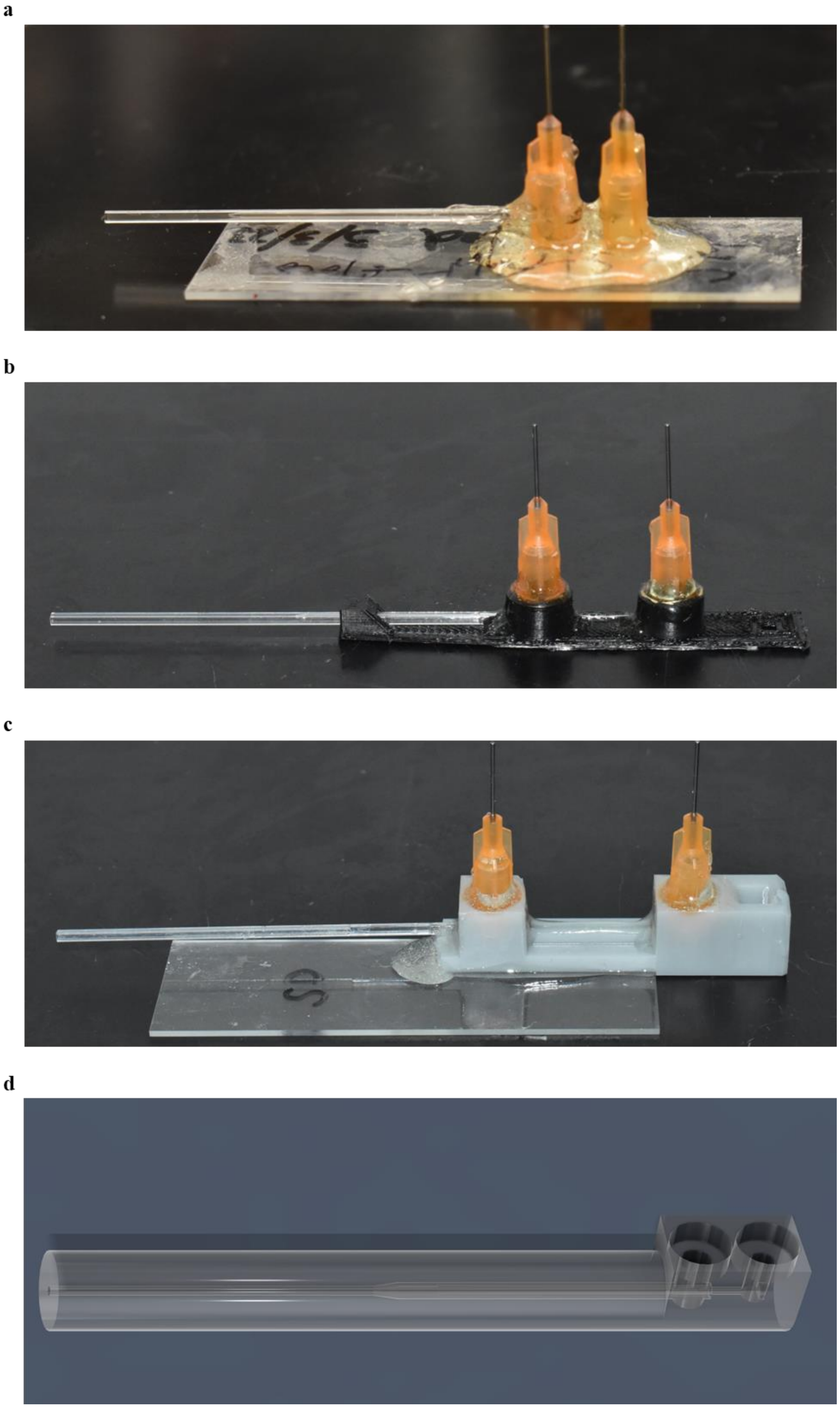

Figure S4. Microfluidic device designs. (a) The initial microfluidic device is fully manually constructed. **(b)** Microfluidic device with PLA blank created with Creality Ender 3 V2 Neo Fused

Filament Fabrication Printer (FFF) using Inland PLA + Black. **(c)** Microfluidic device with resin printed blank created with Elegoo Mars 4 9k Micro Stereolithography mSLA printer using Siraya Tech Grey Fast resin. **(d)** Computer-aided design (CAD) model of an all-in-one microfluidic device.

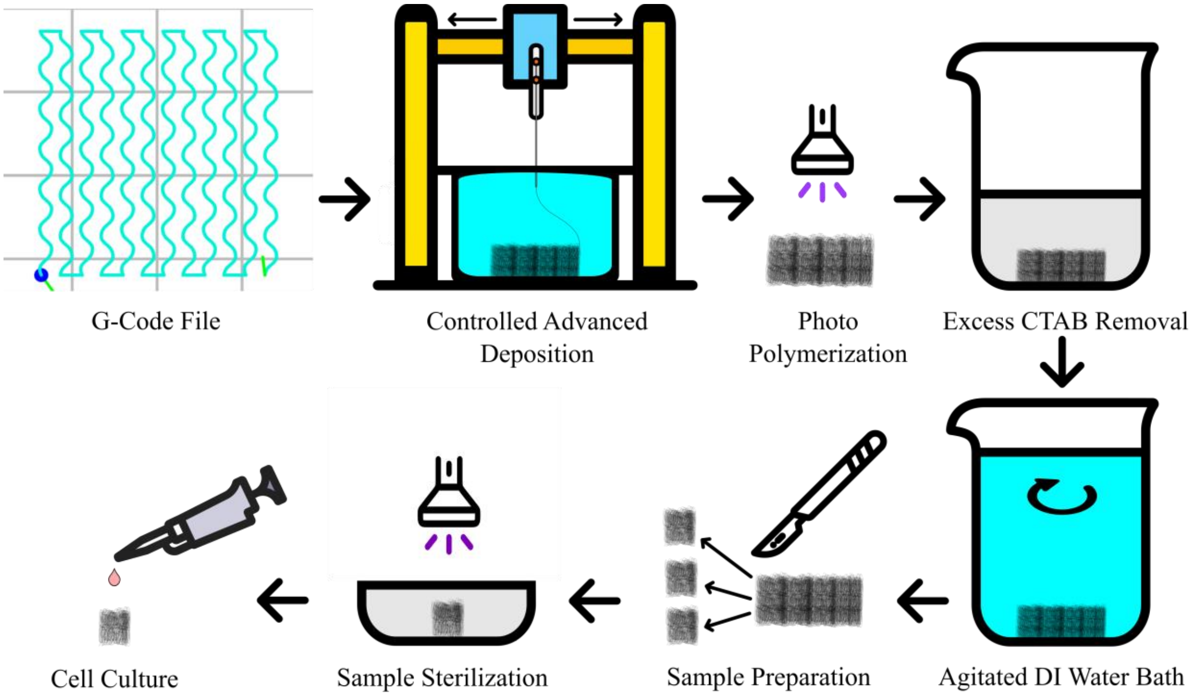

Figure S5. Fabrication of PEGDA-BIPORES multi-fibrous meshwork scaffolds. Schematic representation of the advanced deposition of STrIPS-based BIPORES via g-code mediated precise control of spatial and temporal movements of the extruder head of an Allevi 2 bioprinter, housing the STrIPS-based microfluidic fiber extrusion device. Once the desired amount of deposition was achieved, the 405 nm printer light was utilized for photocrosslinking the fabricated structures. Post-processing of the fabricated scaffolds included excess CTAB removal by 100% (v/v) ethanol treatment, followed by extensive washing in an agitated water bath. The samples were cut into desired dimensions using a scalpel and sterilized using 70% (v/v) ethanol under UV light in a biosafety cabinet. The sterilized PEGDA-BIPORES multi-fibrous meshwork scaffolds were utilized for cytocompatibility studies following standard cell culture protocols.

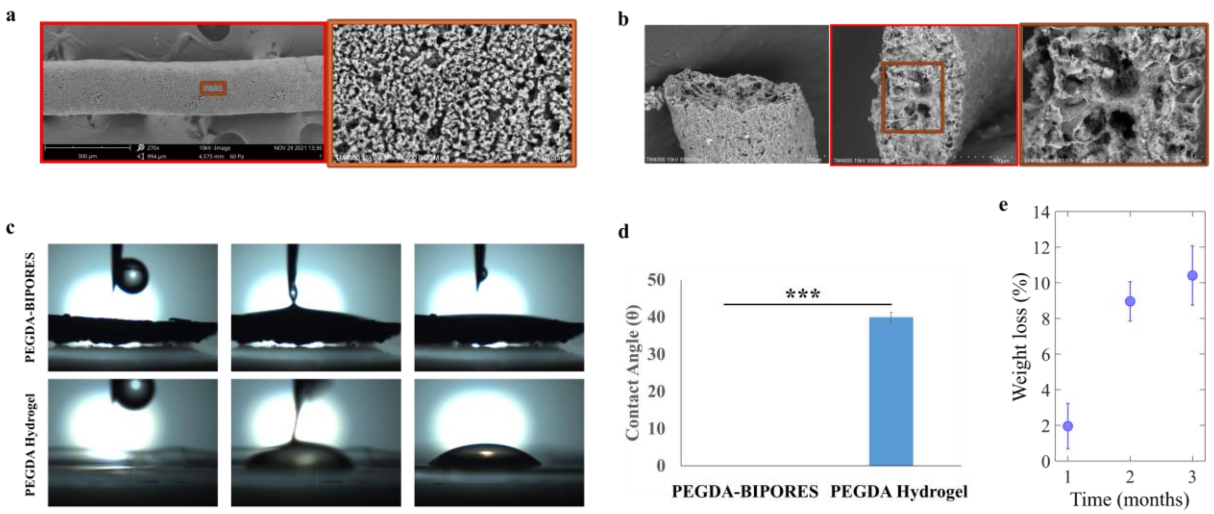

Figure S6. Material Characterization of PEGDA-BIPORES. (a) Scanning electron microscope (SEM) images showing the characteristic bicontinuous structure on the BIPORES fiber surface synthesized at the optimized concentration of nanoparticles and CTAB. A higher magnification surface view of the PEGDA-BIPORES fiber is shown in the red box. **(b)** Representative cross-sectional SEM micrographs showing the bicontinuous porous structure throughout the fiber**. Sessile drop contact angle measurement. (c-d)** PEGDA-BIPORES showed zero contact angle while PEGDA hydrogel showed a contact angle, θ, of about 40°. **(e)** Degradation characteristics of PEGDA-BIPORES fibers in 1X PBS (pH 7.4) inside a cell culture incubator over a period of 3 months. Data are means ± SD. *p*-values were determined by Student's *t*-test (n=3, $^{***}p < 0.001$).

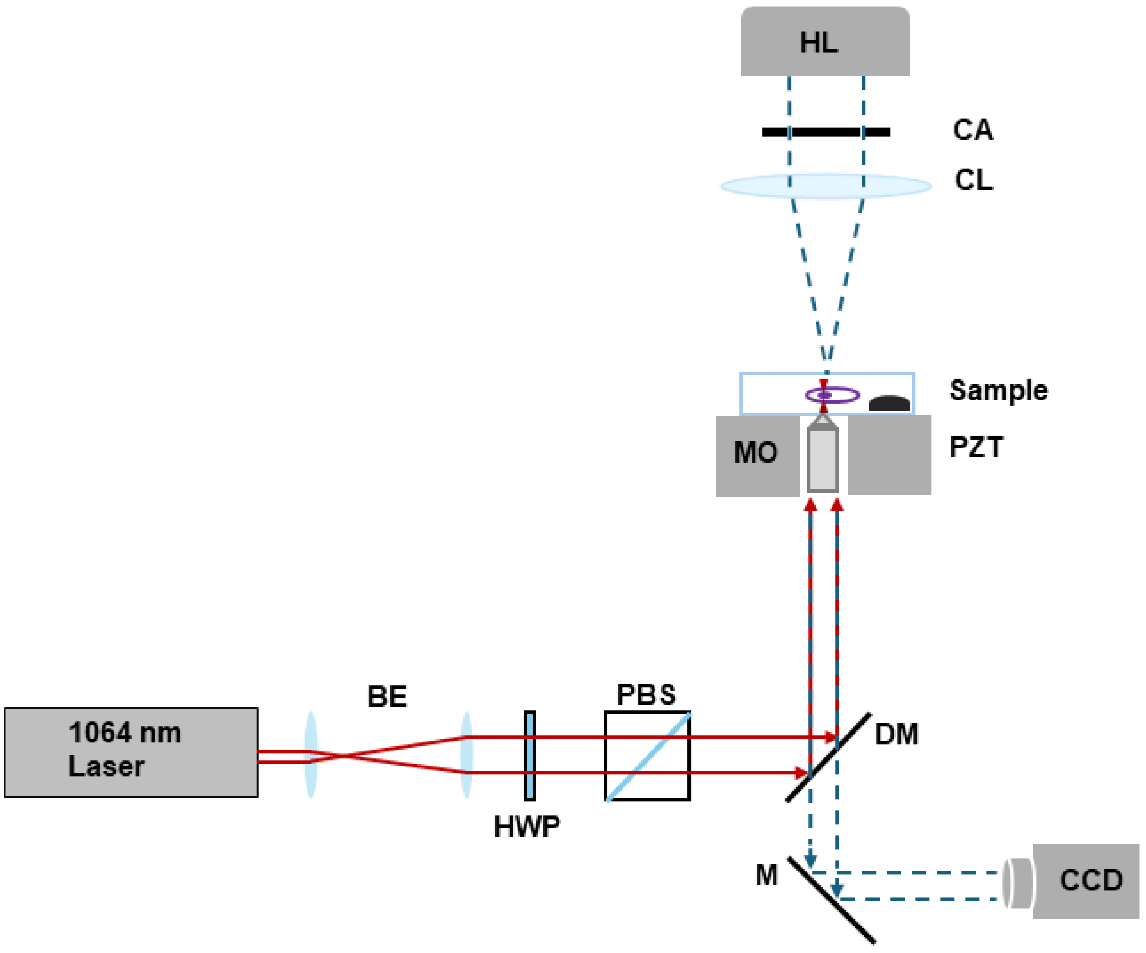

Figure S7. Schematic of optical tweezers system. BE: beam expander, HWP: half-wave plate, PBS: polarizing beam splitter, DM: dichroic mirror, MO: microscope objective, PZT: piezoelectric translation stage, CA: condenser annulus, HL: halogen lamp, M: mirror, and CCD: charge-coupled detector camera. The red path indicates the direction of the laser light for optical trapping, and the blue path indicates the direction of the halogen lamp lighting for imaging.

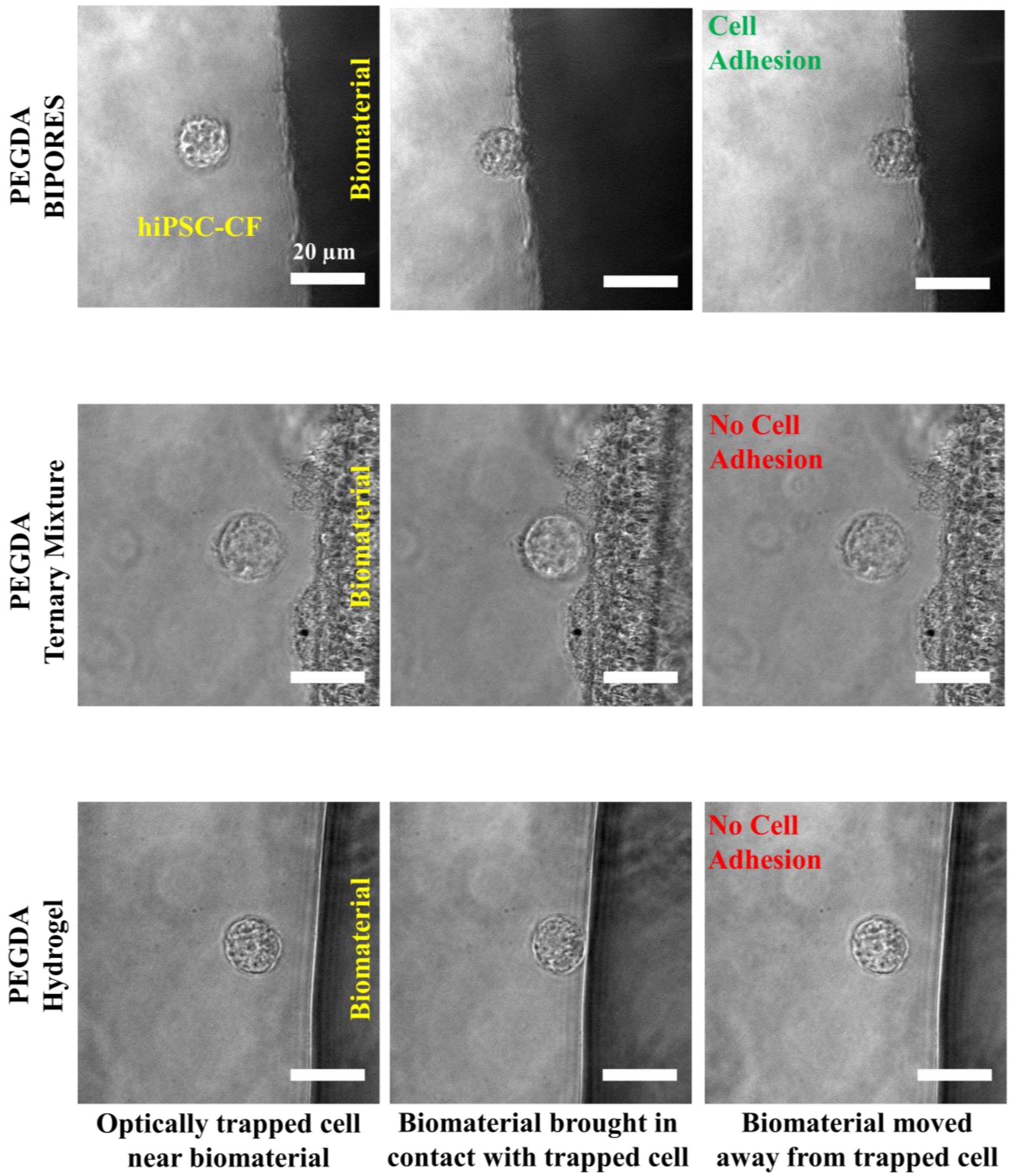

Figure S8. Real-time assessment of single cell-biomaterial interactions. Microscopic images of PEGDA-BIPORES fibers, crosslinked PEGDA ternary mixture, and crosslinked PEGDA hydrogel - near, in contact with, and away from optically trapped human induced pluripotent stem cell (hiPSC)-derived cardiac fibroblast (hiPSC-CF). The cell attached to the PEGDA-BIPORES fibers but failed to attach to the ternary precursor or PEGDA hydrogel scaffolds (scale bar: 20 μm).

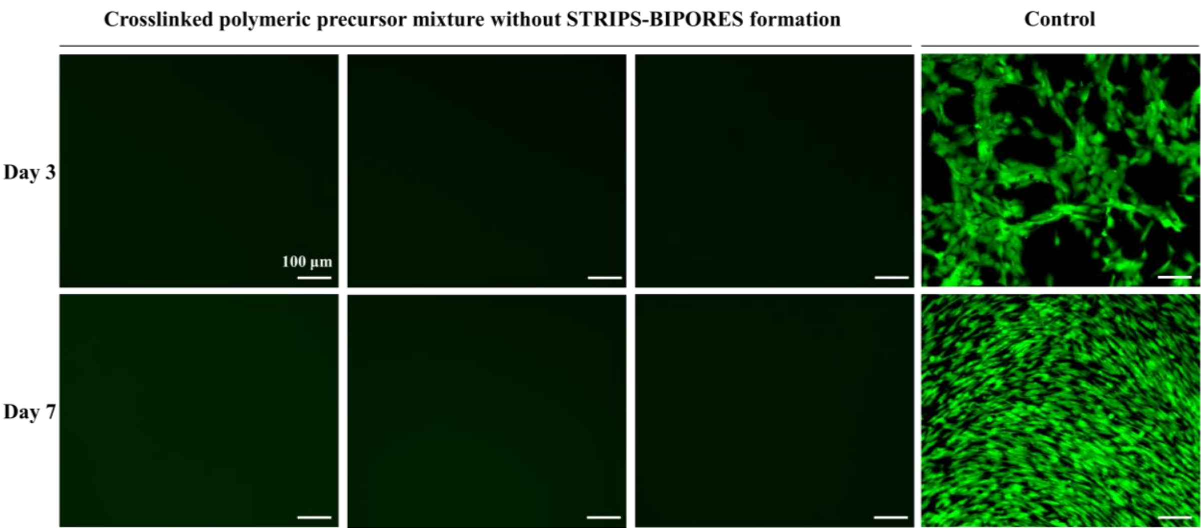

Figure S9. Evaluation of the effect of STrIPS-BIPORES structural characteristics on cytocompatibility of PEGDA. 2D culture of human mesenchymal stem cells (hMSCs) on a scaffold made of crosslinked precursor PEGDA ternary mixture devoid of characteristic STrIPS-BIPORES-associated interconnected porosity at days 3 and 7. The complete lack of cell attachment, growth, and proliferation over time, in comparison to control conditions, underscores the potential significance of the characteristic STrIPS-BIPORES structural features. Viability was evaluated using calcein-AM staining for live cells, represented in green, and ethidium homodimer-I staining for dead cells, depicted in red (scale bar: 100 µm).

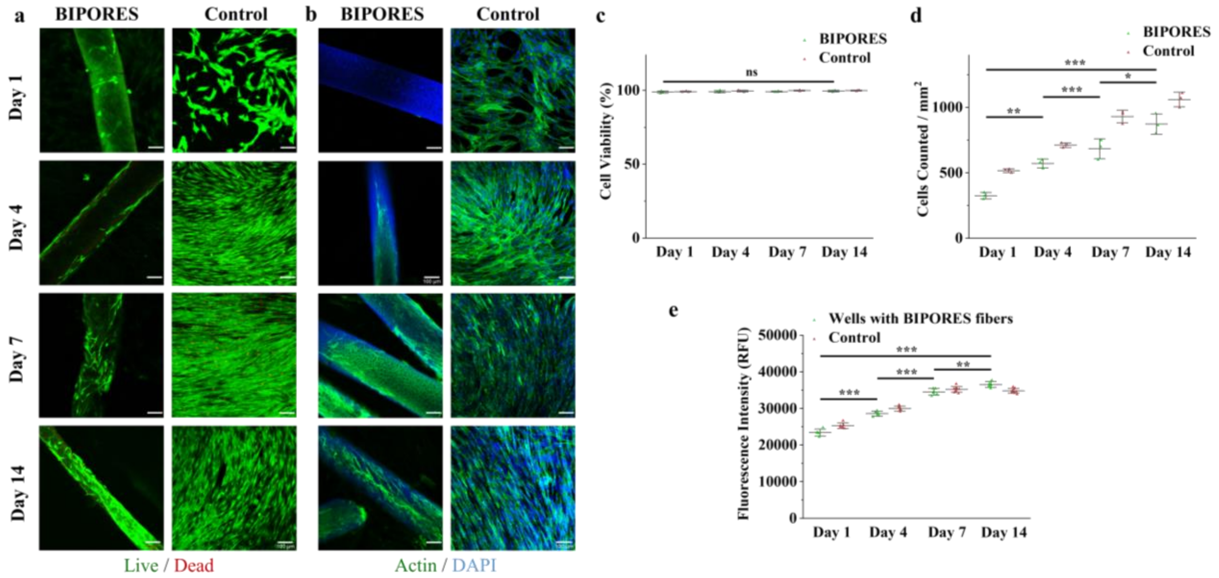

Figure S10. Bioactivity of PEGDA-BIPORES fibers. Fluorescent microscopic imaging of human mesenchymal stem cells (hMSCs) cultured on PEGDA-BIPORES fibers showed good cell proliferation. **(a)** Live and dead cells, stained with calcein-AM (green) and ethidium homodimer-I (red), respectively. **(b)** Phalloidin-stained F-actin (green) and DAPI-stained nuclei (blue) (scale bar: 100 µm). Quantification of **(c)** cell viability based on live and dead cell assay images, **(d)** cell proliferation based on DAPI-stained cell nuclei, and **(e)** fluorescence intensity obtained from PrestoBlue assay of cells on the well and on PEGDA-BIPORES fibers on days 1, 4, 7, and 14 post-cell seeding. Data are means ± SD. *p*-values were determined by one-way ANOVA with Tukey's post-hoc test (n=3 (biological replicates), $^{*}p < 0.05$, $^{**}p < 0.01$, $^{***}p < 0.001$, ns – non-significant).

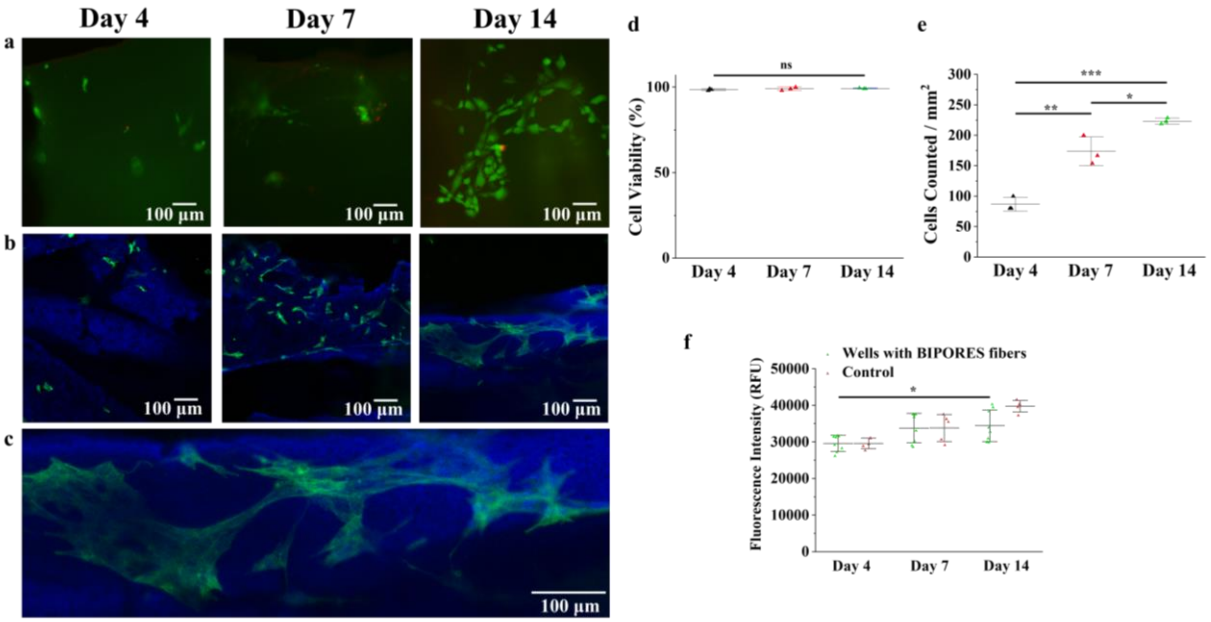

Figure S11. Assessment of cytocompatibility of the PEGDA-BIPORES fibers using C2C12 cells. (a) Live (green) and dead (red) assay of cells on PEGDA-BIPORES fibers on days 4, 7, and 14 post-cell seeding. **(b)** Phalloidin and DAPI staining of immortal mouse myoblast C2C12 cells on PEGDA-BIPORES fibers and **(c)** enlarged view of attached phalloidin & DAPI stained cells on fibers showing the characteristic BIPORES morphology. Quantification of **(d)** cell viability, **(e)** number of cells per square mm of the PEGDA-BIPORES fibers, and **(f)** fluorescence intensity obtained from PrestoBlue assay of cells on the well and fibers on days 4, 7, and 14 post-cell seeding. Data are means ± SD. *P*-values were determined by one-way ANOVA with Tukey's post-hoc test (n=3 (biological replicates), *$p < 0.05$, **$p < 0.01$, ***$p < 0.001$, ns – non-significant).

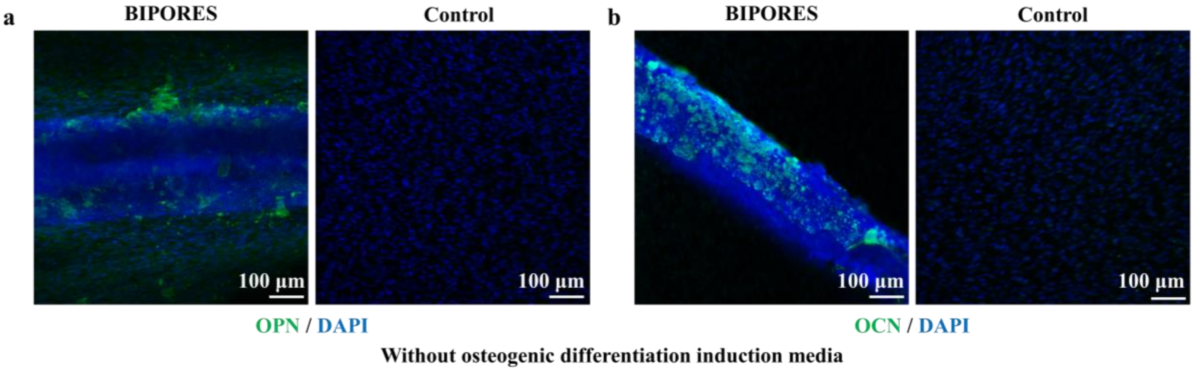

Figure S12. Demonstration of osteogenic differentiation induction potential of PEGDA-BIPORES fibers. Immunofluorescence staining of human mesenchymal stem cells (hMSCs) cultured on PEGDA-BIPORES fibers and tissue culture plate (control) for 21 days in regular hMSC maintenance media composed of DMEM/F-12 supplemented with 15% v/v FBS. Osteogenic differentiation was detected by **(a)** osteopontin (OPN), and **(b)** osteocalcin (OCN), with the nuclei counterstained by DAPI (scale bar: 100 µm).

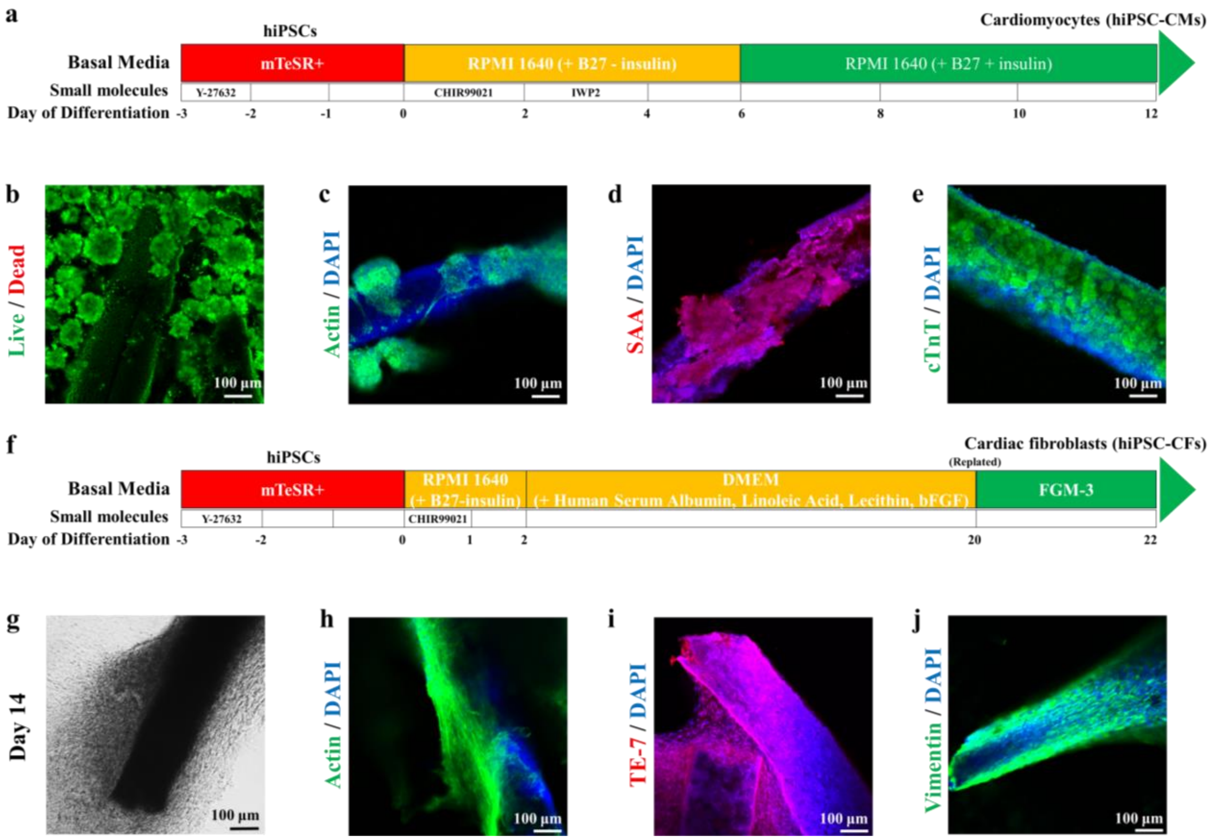

Figure S13. (a) Schematic showing the protocol for differentiating human induced pluripotent stem cells (hiPSCs) into cardiomyocytes (hiPSC-CMs). **Bioactivity of hiPSC-CMs on PEGDA-BIPORES fibers. (b)** Calcein-AM/ethidium homodimer-I based live (green)/dead (red) cell viability assay, **(c)** Actin cytoskeleton and nuclei of hiPSC-CMs on PEGDA-BIPORES fibers stained with Alexa Fluor 488-tagged phalloidin and DAPI, respectively. **(d, e)** Immunofluorescence staining and confocal microscopic imaging of hiPSC-CMs seeded on PEGDA-BIPORES fibers. Beating hiPSC-CMs on the fibers were immunostained for **(d)** sarcomeric α-actinin (SAA), and **(e)** cardiac troponin T (cTnT), with the nuclei counterstained by DAPI, on day 7 post-seeding (scale bar: 100 µm). **(f)** Schematic showing the protocol for differentiating hiPSCs into cardiac fibroblasts (hiPSC-CFs). **Bioactivity of hiPSC-CFs on PEGDA-BIPORES fibers. (g)** Representative brightfield microscopic image showing confluent viable hiPSC-CFs on PEGDA-BIPORES fiber, **(h)** Actin cytoskeleton and nuclei of hiPSC-CFs on PEGDA-BIPORES fibers stained with Alexa Fluor 488-tagged phalloidin and DAPI, respectively. **(i, j)** Immunofluorescence staining and confocal microscopic imaging of hiPSC-CFs seeded on PEGDA-BIPORES fibers. Confluent hiPSC-CFs on the fibers were immunostained for **(i)** TE-7, and **(j)** vimentin, with the nuclei counterstained by DAPI, on day 14 post-seeding (scale bar: 100 µm).

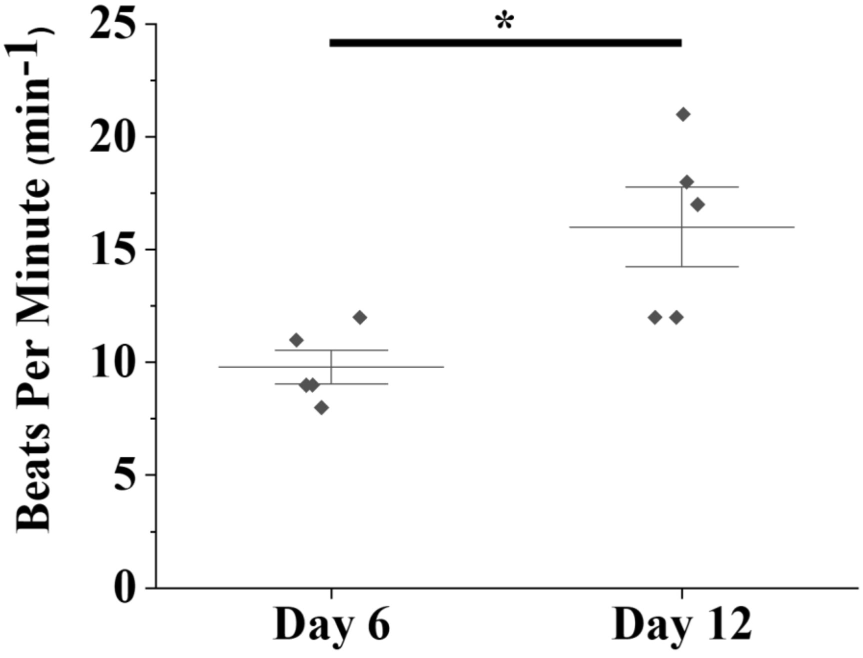

Figure S14. Quantification of beating rate of hiPSC-CMs co-cultured with hiPSC-CFs on PEGDA-BIPORES fibers. Spontaneous beating rate of human induced pluripotent stem cells (hiPSC)-derived cardiomyocytes (hiPSC-CMs) co-cultured with hiPSC-derived cardiac fibroblasts (hiPSC-CFs) at a 4:1 ratio, respectively. The beating rates were calculated from videos recorded on days 6 and 12. Data are means ± SD. *P*-values were determined by Student's *t*-test (n=5 (biological replicates), *$p < 0.05$).

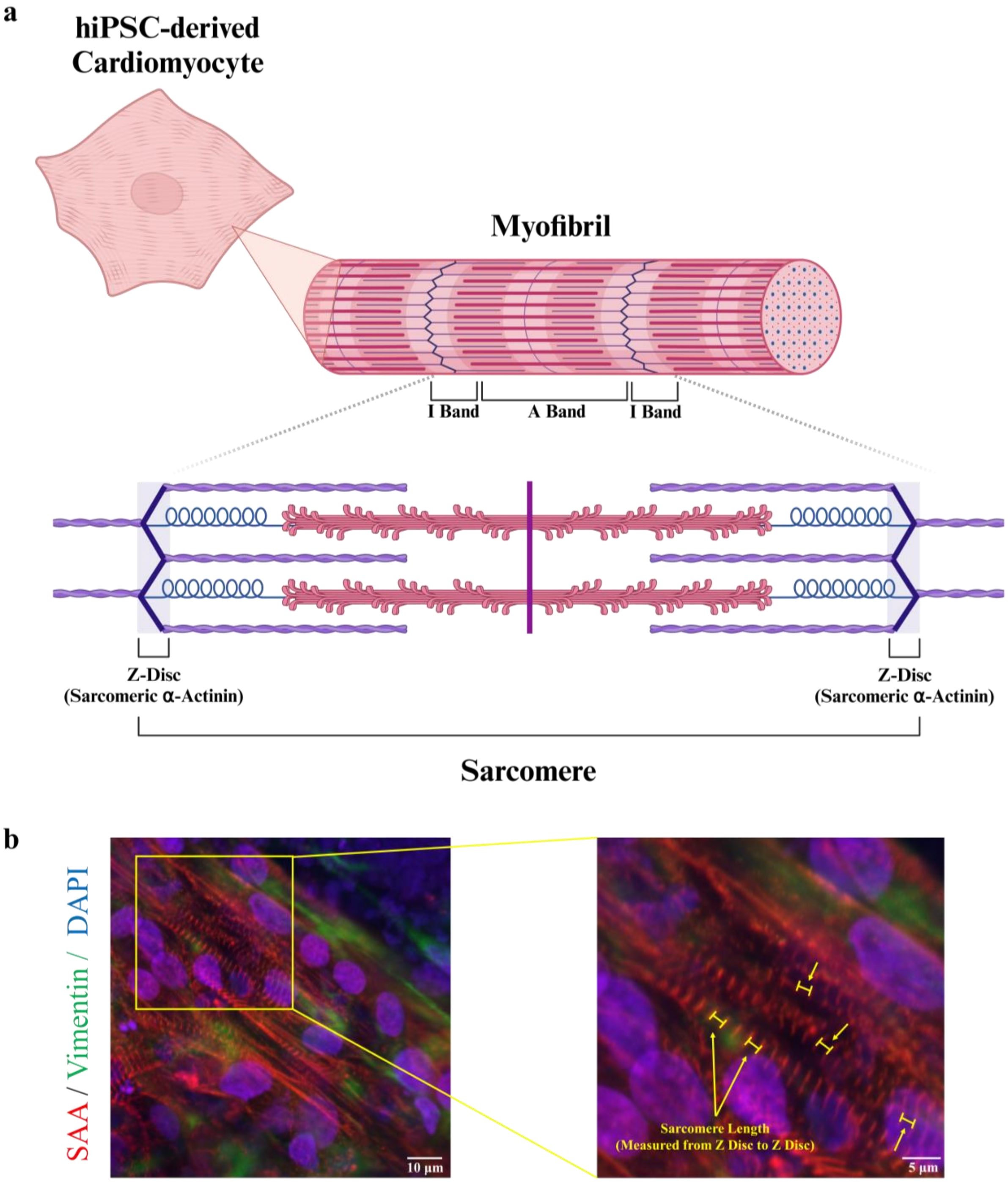

Figure S15. Sarcomere length quantification. (a) Schematic of a sarcomeric unit of a human induced pluripotent stem cell (hiPSC)-derived cardiomyocyte, showing the main structures. The Z-discs are shown in dark blue. In our study, an anti-sarcomeric α-actinin (SAA) antibody was used to stain the Z-discs. **(b)** Representative confocal laser scanning microscopic image showing the SAA staining. The sarcomere lengths were calculated by measuring the distances from Z-disc to Z-disc (Right: yellow arrows and yellow bars).

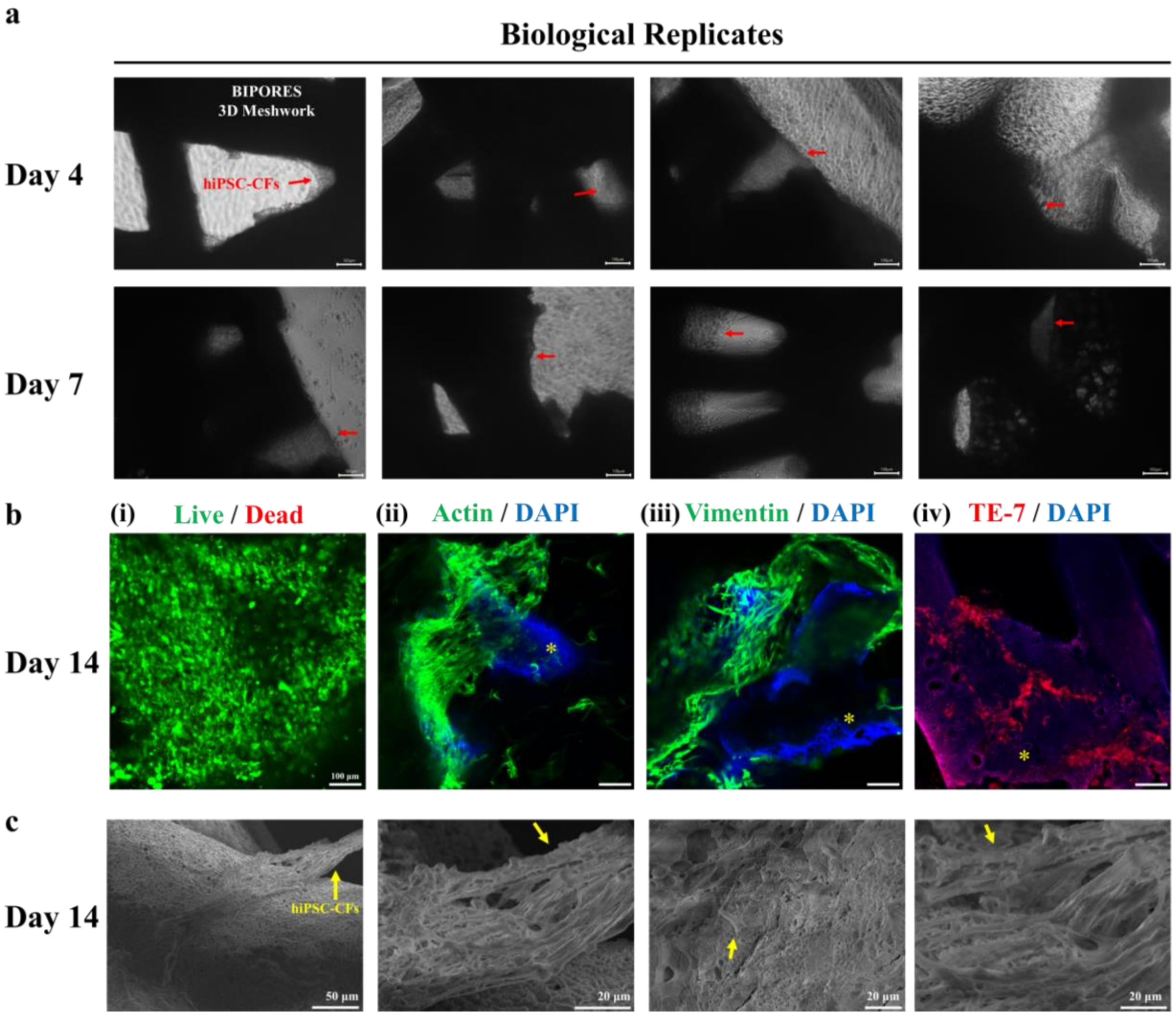

Figure S16. Bioactivity of hiPSC-CFs on PEGDA-BIPORES multi-fibrous 3D meshwork scaffolds. (a) Brightfield microscopic imaging of human induced pluripotent stem cell-derived cardiac fibroblasts (hiPSC-CFs) cultured on PEGDA-BIPORES 3D meshwork scaffolds on days 4 and 7. The optically opaque structures are the 3D meshwork scaffolds. The red arrows indicate a dense layer of hiPSC-CFs formed on the multi-fibrous networks of PEGDA-BIPORES scaffolds (scale bar: 100 µm). **(b) (i)** Representative fluorescence microscopic image showing confluent viable hiPSC-CFs on PEGDA-BIPORES multi-fibrous scaffolds, **(ii)** Actin cytoskeleton and nuclei of hiPSC-CFs on PEGDA-BIPORES scaffolds stained with Alexa Fluor 488-tagged phalloidin and DAPI, respectively. **(iii, iv)** Immunofluorescence staining and confocal microscopic imaging of hiPSC-CFs seeded on PEGDA-BIPORES multi-fibrous meshwork. Confluent hiPSC-CFs on the meshwork were immunostained for **(iii)** vimentin, and **(iv)** anti-fibroblast TE-7, with the nuclei counterstained by DAPI, on day 14 post-seeding (scale bar: 100 µm). The yellow asterisk represents the fibers in focus. **Morphological assessment of cell-laden PEGDA-BIPORES multi-fibrous scaffolds. (c)** Scanning electron microscope (SEM) micrographs of hiPSC-CFs grown on PEGDA-BIPORES fibrous meshwork for 14 days, at different magnifications.

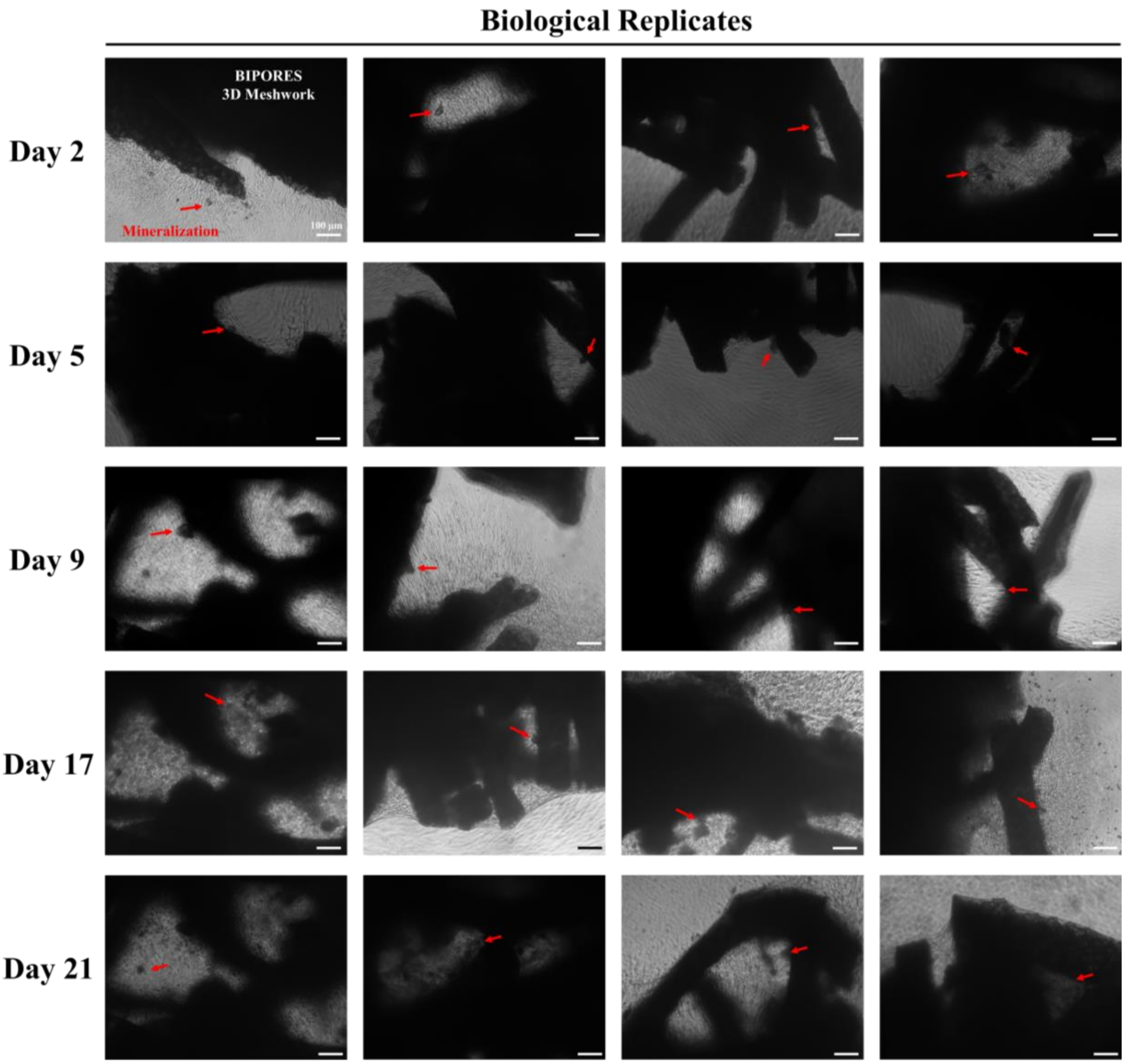

Figure S17. Osteogenic differentiation of hMSCs on PEGDA-BIPORES multi-fibrous 3D meshwork scaffolds. Brightfield microscopic imaging of osteogenically differentiating human mesenchymal stem cells (hMSCs) cultured on PEGDA-BIPORES 3D meshwork scaffolds on days 2, 5, 9, 17, and 21 post-induction of osteogenic differentiation. The optically opaque structures are the 3D meshwork scaffolds. The red arrows indicate mineralization, deposited within the multi-fibrous networks of PEGDA-BIPORES scaffolds (scale bar: 100 μm).

Movie S1. Movie of PEGDA-BIPORES fiber fabrication process.

Movie S2. Movie of optimal fluid flow in a properly aligned fiber extrusion microfluidic device.

Movie S3. Movie of fabrication of PEGDA-BIPORES multi-fibrous meshwork scaffolds.

Movie S4. Movie of single hMSC-PEGDA-BIPORES interaction in tapping mode.

Movie S5. Movie of single hMSC-PEGDA ternary mixture interaction in tapping mode.

Movie S6. Movie of single hMSC-PEGDA hydrogel interaction in tapping mode.

Movie S7. Movie of single hMSC-PEGDA-BIPORES interaction in contact mode.

Movie S8. Movie of single hMSC-PEGDA ternary mixture interaction in contact mode.

Movie S9. Movie of single hMSC-PEGDA hydrogel interaction in contact mode.

Movie S10. Movie of single hiPSC-CF-PEGDA-BIPORES interaction in tapping mode.

Movie S11. Movie of single hiPSC-CF -PEGDA ternary mixture interaction in tapping mode.

Movie S12. Movie of single hiPSC-CF -PEGDA hydrogel interaction in tapping mode.

Movie S13. Movie of single hiPSC-CF -PEGDA-BIPORES interaction in contact mode.

Movie S14. Movie of single hiPSC-CF -PEGDA ternary mixture interaction in contact mode.

Movie S15. Movie of single hiPSC-CF -PEGDA hydrogel interaction in contact mode.

Movies S16-17. Movie of beating hiPSC-CMs on PEGDA-BIPORES fibers.

Movies S18-19. Movie of beating hiPSC-CMs, co-cultured with hiPSC-CFs, on PEGDA-BIPORES fibers.

Movie S20. Movie of beating hiPSC-CMs, co-cultured with hiPSC-CFs, on PEGDA-BIPORES multi-fibrous meshwork scaffolds.
